# Prognostic risk factors for moderate-to-severe exacerbations in patients with chronic obstructive pulmonary disease: a systematic literature review

**DOI:** 10.1186/s12931-022-02123-5

**Published:** 2022-08-23

**Authors:** John R. Hurst, MeiLan K. Han, Barinder Singh, Sakshi Sharma, Gagandeep Kaur, Enrico de Nigris, Ulf Holmgren, Mohd Kashif Siddiqui

**Affiliations:** 1 UCL Respiratory, University College London, London, WC1E 6BT UK; 2 Division of Pulmonary and Critical Care, University of Michigan, Ann Arbor, MI USA; 3 Formerly of Parexel International, Mohali, India; 4 Parexel International, Mohali, India; 5 Formerly of AstraZeneca, Cambridge, UK; 6 AstraZeneca, Gothenburg, Sweden

**Keywords:** Exacerbations, COPD, Systematic literature review, Comorbidities, Hospitalization, Predictors, Biomarkers

## Abstract

**Background:**

Chronic obstructive pulmonary disease (COPD) is a leading cause of morbidity and mortality worldwide. COPD exacerbations are associated with a worsening of lung function, increased disease burden, and mortality, and, therefore, preventing their occurrence is an important goal of COPD management. This review was conducted to identify the evidence base regarding risk factors and predictors of moderate-to-severe exacerbations in patients with COPD.

**Methods:**

A literature review was performed in Embase, MEDLINE, MEDLINE In-Process, and the Cochrane Central Register of Controlled Trials (CENTRAL). Searches were conducted from January 2015 to July 2019. Eligible publications were peer-reviewed journal articles, published in English, that reported risk factors or predictors for the occurrence of moderate-to-severe exacerbations in adults age ≥ 40 years with a diagnosis of COPD.

**Results:**

The literature review identified 5112 references, of which 113 publications (reporting results for 76 studies) met the eligibility criteria and were included in the review. Among the 76 studies included, 61 were observational and 15 were randomized controlled clinical trials. Exacerbation history was the strongest predictor of future exacerbations, with 34 studies reporting a significant association between history of exacerbations and risk of future moderate or severe exacerbations. Other significant risk factors identified in multiple studies included disease severity or bronchodilator reversibility (39 studies), comorbidities (34 studies), higher symptom burden (17 studies), and higher blood eosinophil count (16 studies).

**Conclusions:**

This systematic literature review identified several demographic and clinical characteristics that predict the future risk of COPD exacerbations. Prior exacerbation history was confirmed as the most important predictor of future exacerbations. These prognostic factors may help clinicians identify patients at high risk of exacerbations, which are a major driver of the global burden of COPD, including morbidity and mortality.

**Supplementary Information:**

The online version contains supplementary material available at 10.1186/s12931-022-02123-5.

## Background

Chronic obstructive pulmonary disease (COPD) is the third leading cause of death worldwide [1]. Based upon disability-adjusted life-years, COPD ranked sixth out of 369 causes of global disease burden in 2019 [2]. COPD exacerbations are associated with a worsening of lung function, and increased disease burden and mortality (of those patients hospitalized for the first time with an exacerbation, > 20% die within 1 year of being discharged) [3]. Furthermore, patients with COPD consider exacerbations or hospitalization due to exacerbations to be the most important disease outcome, having a large impact on their lives [4]. Therefore, reducing the future risk of COPD exacerbations is a key goal of COPD management [5].

Being able to predict the level of risk for each patient allows clinicians to adapt treatment and patients to adjust their lifestyle (e.g., through a smoking cessation program) to prevent exacerbations [3]. As such, identifying high-risk patients using measurable risk factors and predictors that correlate with exacerbations is critical to reduce the burden of disease and prevent a cycle of decline encompassing irreversible lung damage, worsening quality of life (QoL), increasing disease burden, high healthcare costs, and early death.

Prior history of exacerbations is generally thought to be the best predictor of future exacerbations; however, there is a growing body of evidence suggesting other demographic and clinical characteristics, including symptom burden, airflow obstruction, comorbidities, and inflammatory biomarkers, also influence risk [6–9]. For example, in the prospective ECLIPSE observational study, the likelihood of patients experiencing an exacerbation within 1 year of follow-up increased significantly depending upon several factors, including prior exacerbation history, forced expiratory volume in 1 s (FEV_1_), St. George’s Respiratory Questionnaire (SGRQ) score, gastroesophageal reflux, and white blood cell count [9].

Many studies have assessed predictors of COPD exacerbations across a variety of countries and patient populations. This systematic literature review (SLR) was conducted to identify and compile the evidence base regarding risk factors and predictors of moderate-to-severe exacerbations in patients with COPD.

## Methods

### Systematic literature review

A comprehensive search strategy was designed to identify English-language studies published in peer-reviewed journals providing data on risk factors or predictors of moderate or severe exacerbations in adults aged ≥ 40 years with a diagnosis of COPD (sample size ≥ 100). The protocol is summarized in Table 1 and the search strategy is listed in Additional file 1: Table S1. Key biomedical electronic literature databases were searched from January 2015 until July 2019. Other sources were identified via bibliographic searching of relevant systematic reviews.

**Table 1:** Summary of the protocol for the systematic literature review

Criteria	Inclusion criteria
Objective and research question	• To identify the evidence related to COPD exacerbations, including literature on risk factors and predictors of exacerbations
Studies to include	
Study design	• All studies providing data on risk of exacerbations, including clinical trials and real-world observational studies • Published in peer-reviewed journals
Population	• Age: ≥ 40 years • Sex: any • Race: any • Disease: COPD • Sample size: ≥ 100 • Exacerbation type: moderate or severe
Interventions	• No restriction
Comparators	• No restriction
Language	• English
Publication time frame	• January 2015–July 2019
Data sources	
Databases	• Embase • MEDLINE • MEDLINE In-Process • Cochrane Central Register of Controlled Trials (CENTRAL)
Other	• Bibliographic searching using relevant systematic literature reviews

*COPD* chronic obstructive pulmonary disease

### Study selection process

Implementation and reporting followed the recommendations and standards of the Preferred Reporting Items for Systematic reviews and Meta-analyses (PRISMA) statement [10]. An independent reviewer conducted the first screening based on titles and abstracts, and a second reviewer performed a quality check of the excluded evidence. A single independent reviewer also conducted the second screening based on full-text articles, with a quality check of excluded evidence performed by a second reviewer. Likewise, data tables of the included studies were generated by one reviewer, and another reviewer performed a quality check of extracted data. Where more than one publication was identified describing a single study or trial, data were compiled into a single entry in the data-extraction table to avoid double counting of patients and studies. One publication was designated as the ‘primary publication’ for the purposes of the SLR, based on the following criteria: most recently published evidence and/or the article that presented the majority of data (e.g., journal articles were preferred over conference abstracts; articles that reported results for the full population were preferred over later articles providing results of subpopulations). Other publications reporting results from the same study were designated as ‘linked publications’; any additional data in the linked publications that were not included in the primary publication were captured in the SLR. Conference abstracts were excluded from the SLR unless they were a ‘linked publication.’

## Results

### Included studies

**Fig. 1:**
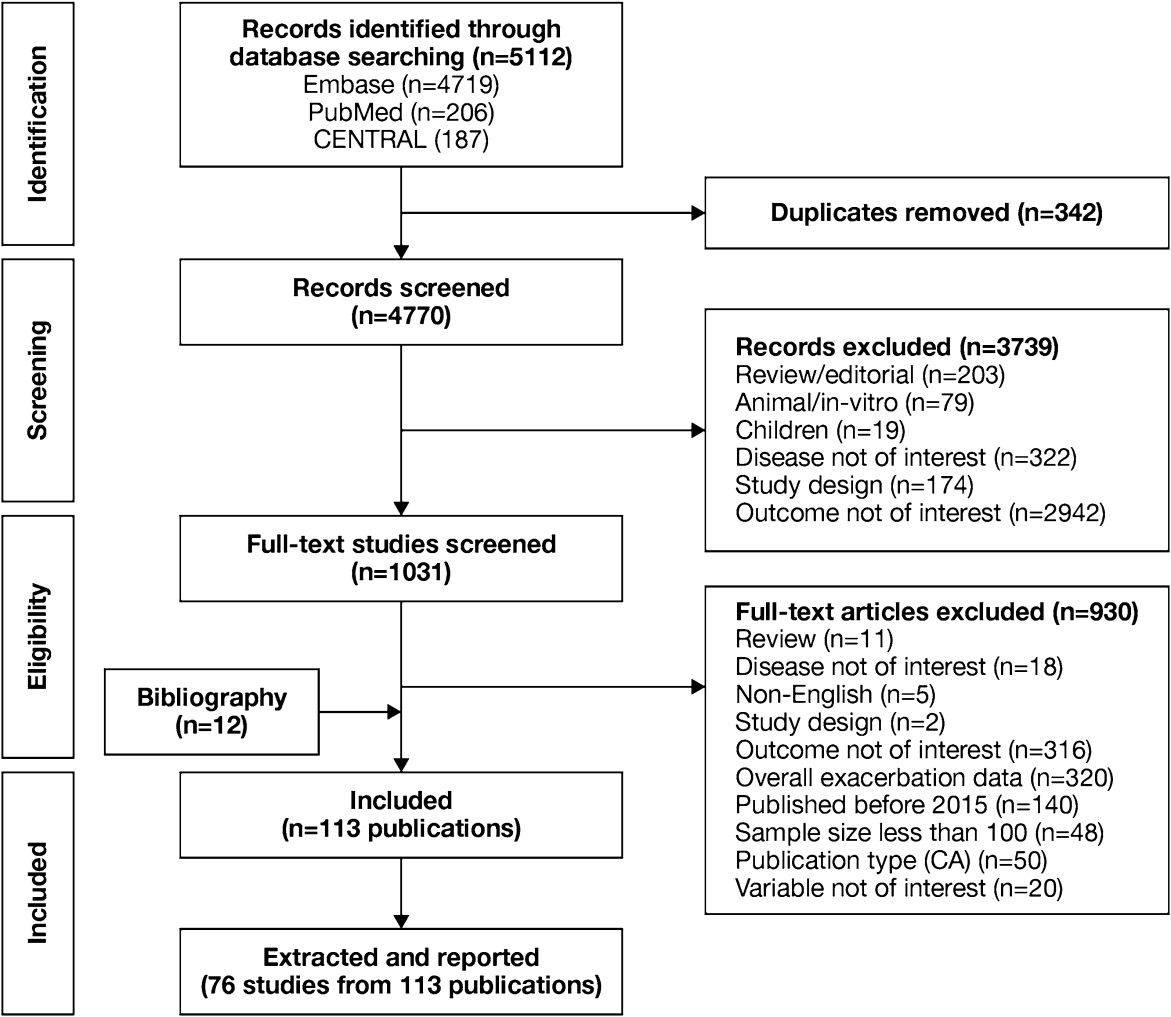
PRISMA flow diagram of studies through the systematic review process. *CA* conference abstract, *CENTRAL* Cochrane Central Register of Controlled Trials, *PRISMA* Preferred Reporting Items for Systematic Reviews and Meta-Analyses

A total of 5112 references (Fig. 1) were identified from the database searches. In total, 76 studies from 113 publications were included in the review. Primary publications and ‘linked publications’ for each study are detailed in Additional file 1: Table S2, and study characteristics are shown in Additional file 1: Table S3. The studies included clinical trials, registry studies, cross-sectional studies, cohort studies, database studies, and case–control studies. All 76 included studies were published in peer-reviewed journals. Regarding study design, 61 of the studies were observational (34 retrospective observational studies, 19 prospective observational studies, four cross-sectional studies, two studies with both retrospective and prospective cohort data, one case–control study, and one with cross-sectional and longitudinal data) and 15 were randomized controlled clinical trials.

Of the 76 studies, 16 were conducted in North America (13 studies in the USA, two in Canada, and one in Mexico); 26 were conducted in Europe (seven studies in Spain, four in the UK, three in Denmark, two studies each in Bulgaria, the Netherlands, and Switzerland, and one study each in Sweden, Serbia, Portugal, Greece, Germany, and France) and 17 were conducted in Asia (six studies in South Korea, four in China, three in Taiwan, two in Japan, and one study each in Singapore and Israel). One study each was conducted in Turkey and Australia. Fifteen studies were conducted across multiple countries.

The majority of the studies (n = 54) were conducted in a multicenter setting, while 22 studies were conducted in a single-center setting. The sample size among the included studies varied from 118 to 339,389 patients.

### Patient characteristics

A total of 75 studies reported patient characteristics (Additional file 1: Table S4). The mean age was reported in 65 studies and ranged from 58.0 to 75.2 years. The proportion of male patients ranged from 39.7 to 97.6%. The majority of included studies (85.3%) had a higher proportion of males than females.

Exacerbation history (as defined per each study) was reported in 18 of 76 included studies. The proportion of patients with no prior exacerbation was reported in ten studies (range, 0.1–79.5% of patients), one or fewer prior exacerbation in ten studies (range, 46–100%), one or more prior exacerbation in eight studies (range, 18.4–100%), and two or more prior exacerbations in 12 studies (range, 6.1–55.0%).

### Prognostic factors of exacerbations

**Fig. 2:**
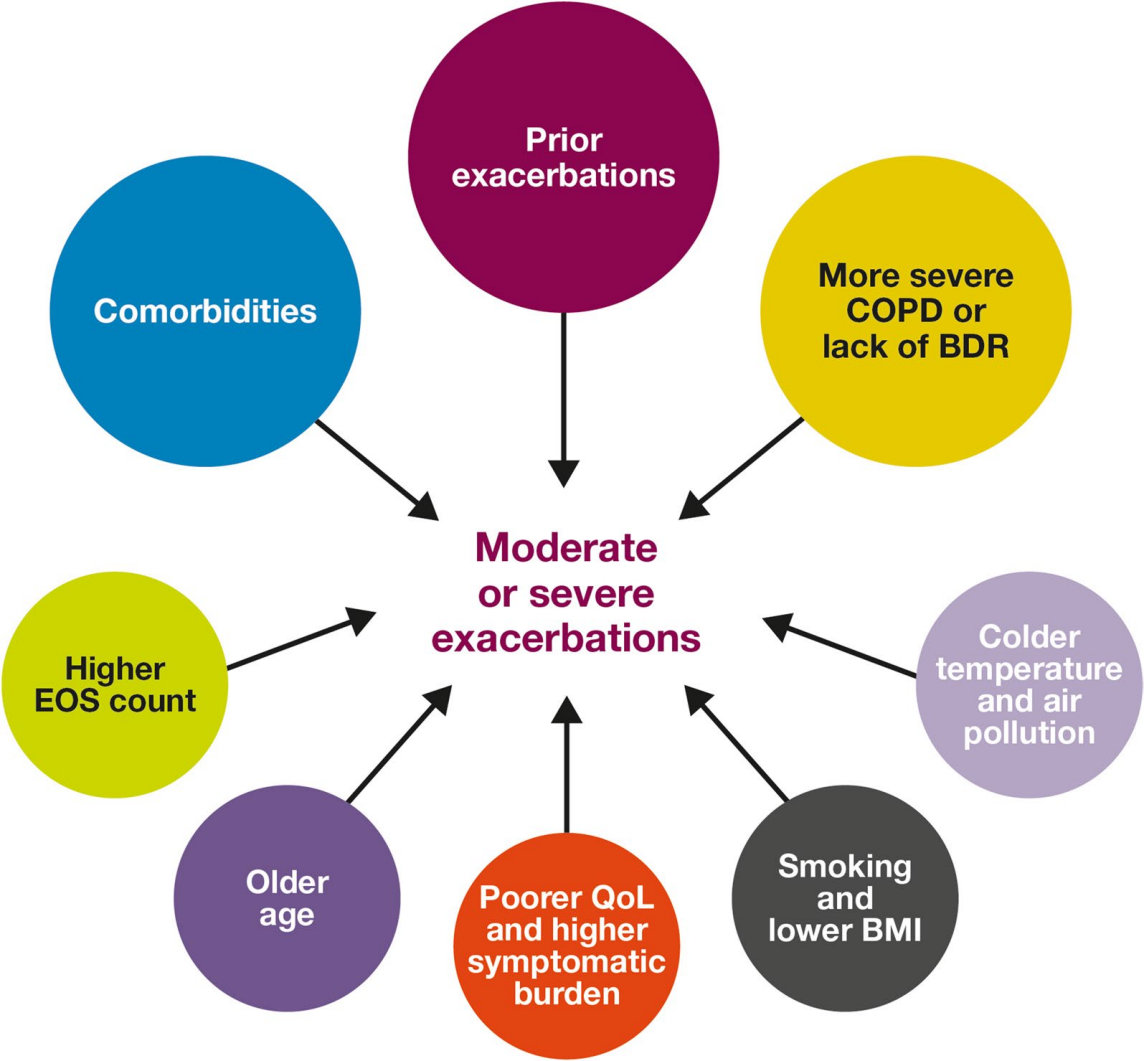
Risk factors for moderate-to-severe exacerbations in patients with COPD. Factors with > 30 supporting studies shown as large circles; factors with ≤ 30 supporting studies shown as small circles and should be interpreted cautiously. *BDR* bronchodilator reversibility, *BMI* body mass index, *COPD* chronic obstructive pulmonary disease, *EOS* eosinophil, *QoL* quality of life

**Fig. 3:**
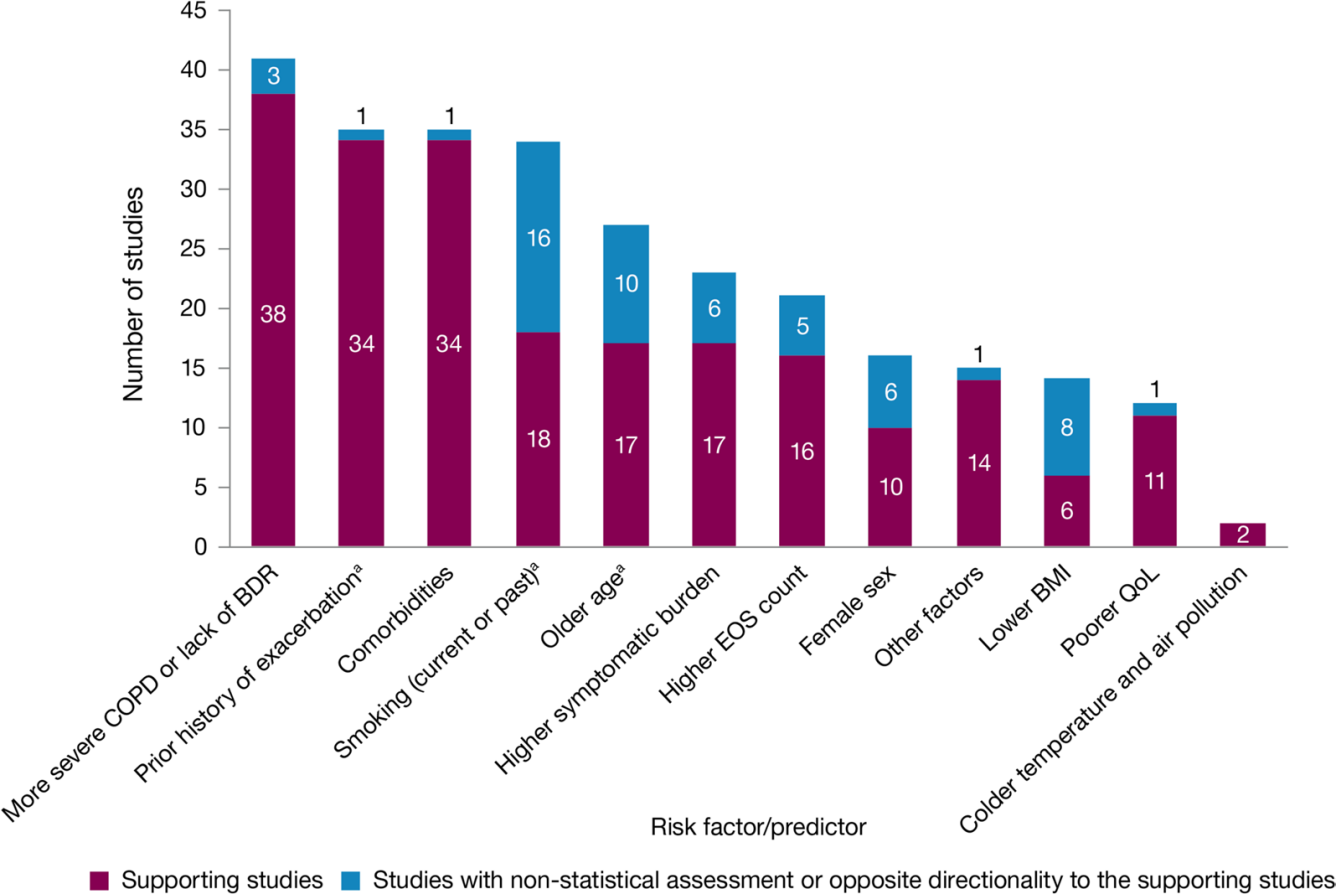
Summary of risk factors for exacerbation events. ^a^Treatment impact studies removed. *BDR* bronchodilator reversibility, *BMI* body mass index, *COPD* chronic obstructive pulmonary disease, *EOS* eosinophil, *QoL* quality of life

A summary of the risk factors and predictors reported across the included studies is provided in Tables 2 and 3. The overall findings of the SLR are summarized in Figs. 2 and 3.

**Table 2:** Summary of risk factors for exacerbation events

Risk factor/predictor	Description
Prior history of exacerbation	The strongest risk factor for future exacerbations is a history of exacerbations within the last 12 months
Comorbidities	Underlying comorbid diseases including anxiety and depression, asthma, blindness and low vision, dyspepsia, heart failure, hypertension, lung cancer, osteoarthritis, peripheral vascular disease, and prostate disorders are associated with increased risk of exacerbations
COPD severity and BDR	The risk of exacerbation is significantly higher in patients with severe or very severe airflow limitation and lack of BDR
Eosinophil count	Higher eosinophil count is associated with an increased risk of exacerbations
Quality of life	Poor quality of life at baseline or worsening quality of life over time (measured by SGRQ, CCQ, and CES-D) are associated with an increase in exacerbation risk
Symptomatic burden	Higher symptomatic burden of COPD (CAT ≥ 10 and mMRC ≥ 2) is associated with an increased risk of exacerbations
Smoking	Smoking (former/current) is associated with an increased risk of exacerbations
BMI	Underweight patients (BMI < 18.5 kg/m^2^) are at higher risk of exacerbations
Age	Older age is associated with an increased risk of exacerbations
Sex	Associations between sex and the risk of exacerbations are variable
Temperature and pollution	Colder temperature and air pollution (NO_2_, O_3_, CO, and PM10) are associated with an increased risk of exacerbations
Other factors	Low physical activity (decreased 6MWD), elevated inflammatory biomarkers (e.g. C-reactive protein), and certain race/ethnicity/region factors may be associated with an increased risk of exacerbations

*6MWD* six-minute walk distance, *BDR* bronchodilator reversibility, *BMI* body mass index, *CAT* COPD Assessment Test, *CCQ* Clinical COPD Questionnaire, *CES-D* Center for Epidemiological Studies—Depression, *CO* carbon monoxide, *COPD* chronic obstructive pulmonary disease, *FEV*_*1*_ forced expiratory volume in 1 s, *ICS* inhaled corticosteroid, *mMRC* modified Medical Research Council, *NO*_*2*_ nitrogen dioxide, *O*_*3*_ ozone, *PM10* particulate matter ≤ 10 μm in diameter, *SGRQ* St. George’s Respiratory Questionnaire

**Table 3:** Prognostic factors for exacerbations reported across the included studies

Study	Country	Age	Sex	CAT score	mMRC score	Smoking	Temperature and pollution	Prior exacerbation	BMI	6MWD	QoL score	Race, ethnicity, or region	Comorbidities	Eosinophil count	Biomarkers	Disease severity or BDR
Adir 2018 [62]	Israel	–	–	–	–	–	–	–	–	–	–	–	–	✔	–	**✔**^**a**^
Alexopoulos 2015 (GOLDEN study) [28]	Greece	**✔**	**✔**	–	–	**✔**	–	–	**✔**	–	–	–	**✔**	–	–	**✔**^**b**^
Annavarapu 2018 [34]	US	–	–	–	–	**✔**	–	**✔**	–	–	–	**✔**	**✔**	–	–	–
Bade 2019 [80]	US	**✔**	✔	–	–	✔	–	–	–	–	–	**✔**	**✔**	–	–	–
Bafadhel 2018 [37]	Global	–	**✔**	–	–	**✔**	–	**✔**	–	–	–	–	–	✔	–	**✔**
Bartels 2018 [63]	Canada	**✔**	✔	–	–	**✔**	–	–	–	–	–	–	**✔**	–	–	**✔**^**a**^
Baumeler 2016 (PROMISE-COPD study) [33]	EU	✔	–	–	**✔**	✔	–	–	–	**✔**	**✔**	–	**✔**	–	–	**✔**^**a**^
Calverley 2018 (TIOSPIR study) [38]	Global	–	–	–	–	–	–	**✔** [39]	–	–	–	–	–	–	–	–
Chapman 2018 (SUNSET trial) [89]	Global	–	–	–	–	–	–	–	–	–	–	–	–	**✔**	–	–
Couillard 2017 [90]	Canada	–	–	–	–	–	–	–	–	–	–	–	–	**✔**	–	–
Crisafulli 2015 [35]	Spain	–	–	–	–	–	–	**✔**	–	–	–	–	**✔**	–	**✔**	–
de Miguel-Díez 2019 [31]	Spain	–	–	–	–	–	**✔**	–	–	–	–	–	–	–	–	–
Eklöf 2020 (e-publication, 2019) [40]	Denmark	**✔**	✔	–	**✔**	**✔**	–	**✔**	**✔**	–	–	–	**✔**	–	–	**✔**^**a**^
Emura 2019 [66]	Japan	–	–	–	–	–	–	–	–	–	–	–	–	–	–	**✔**^**b**^
Engel 2017 (REDUCE study) [24]	Switzerland	✔	✔	–	**✔**	**✔**	–	–	–	–	–	–	✔	–	–	**✔**
Erol 2018 [67]	Turkey	–	–	–	–	–	–	–	–	–	–	–	–	–	–	✔
Estirado 2018 [41]	Spain	–	–	–	–	**✔**	–	**✔**^**c**^	–	–	–	–	**✔**	–	–	–
Ferguson 2018 (KRONOS study) [91]	Global	–	–	–	–	–	–	–	–	–	–	–	–	**✔**	–	–
Fuhrman 2017 [42]	France	**✔**	**✔**	–	–	–	–	**✔**	–	–	**✔**	–	–	–	–	–
Han 2017 (SPIROMICS study) [20]	US	✔	**✔**	**✔**	–	**✔** [110]	–	**✔**	–	✔	–	✔	**✔** [82]	✔	**✔**	**✔**^**a**^
Han 2018 [68]	Taiwan	–	–	–	–	–	–	–	–	–	–	–	–	–	–	**✔**
Huang 2018 [69]	Taiwan	–	–	–	–	–	–	–	–	–	–	–	–	–	–	**✔**^**b**^
Jo 2018 [18]	South Korea	✔	✔	**✔**	✔	–	–	**✔**	–	–	✔	–	–	–	–	✔
Jo 2018b [27]	South Korea	**✔**	**✔**	–	–	✔	–	–	**✔**	–	–	–	**✔**	–	–	–
Jung 2015 [70]	South Korea	–	–	–	–	–	–	–	–	–	**✔**	–	–	–	–	**✔**^**a**^
Kim 2017 [71]	South Korea	–	–	–	–	–	–	–	–	–	–	–	–	–	–	**✔**
Kim 2019 (COPDGene study) [64]	US	–	–	–	–	–	–	–	–	–	**✔** [81]	–	**✔** [81]	–	–	**✔** [65]
Ko 2019 [92]	China	–	–	–	–	–	–	–	–	–	–	–	–	✔	–	–
Kobayashi 2018 [72]	Japan	–	–	–	–	–	–	–	–	–	–	–	–	–	–	**✔**^**b**^
Krachunov 2017 [32]	Bulgaria	–	–	–	–	–	**✔**	–	–	–	–	–	–	–	–	–
Krachunov 2018 [43]	Bulgaria	–	–	–	**✔**	–	–	**✔**	–	–	–	–	–	–	–	✔^**b**^
Lau 2017 [83]	US	**✔**	**✔**	–	–	–	–	–	–	–	–	**✔**	**✔**	–	–	–
Lee 2019 [73] (Yoon 2019)	Korea	✔	**✔**	**✔**	–	✔	–	–	–	✔	**✔**	–	**✔**	–	**✔**	**✔**
Liu 2015 [29]	China	**✔**	✔	✔	✔	–	–	**✔**	✔	–	**✔**	–	–	–	–	✔^**b**^
MacDonald 2019 [93]	Australia	–	–	–	–	–	–	–	–	–	–	–	–	**✔**	–	–
Make 2015 [44]	Global	✔	**✔**	–	✔	✔	–	**✔**	✔	–	**✔**	–	**✔**	–	–	**✔**
Marçôa 2018 [19]	Portugal	–	–	**✔**	**✔**	–	–	**✔**	–	–	–	–	**✔**	–	–	**✔**
Margüello 2016 [23]	Spain	**✔**	✔	–	–	**✔**	–	**✔**	✔	–	–	–	–	–	–	**✔**
McGarvey 2015 [22]	UK	**✔**	**✔**	–	**✔**	**✔**	–	**✔**	–	–	–	–	**✔**	–	–	**✔**^**a**^
Montserrat-Capdevila 2015 [45]	Spain	**✔**	**✔**	–	–	**✔**	–	**✔**	–	–	–	–	**✔**	–	–	**✔**^**b**^
Montserrat-Capdevila 2016 [46]	Spain	–	**✔**	–	–	**✔**	–	**✔**	–	–	–	–	**✔**	–	–	**✔**^**b**^
Müllerová 2019 (HO-17–18,395 study) [94]	US	–	–	–	–	–	–	–	–	–	–	–	–	**✔**	–	–
Orea-Tejeda 2018 [47]	Mexico	✔	✔	–	–	–	–	**✔**	–	–	–	–	**✔**	–	–	–
Papi 2018 (TRIBUTE trial) [48]	Global	–	**✔**	–	–	**✔**	–	**✔**	–	–	–	–	–	**✔**	–	**✔**
Pascoe 2019 (IMPACT trial) [13]	Global	–	–	–	–	**✔**^**c**^	–	**✔**^**c**^ [49]	–	–	–	–	–	**✔**^**c**^	–	–
Pasquale 2016 [50]	US	–	–	**✔**	✔	–	–	**✔**	–	–	–	–	–	–	–	**✔**
Pavlovic 2017 [74]	Serbia	**✔**	✔	–	–	✔	–	–	✔	–	–	–	**✔**	–	**✔**	**✔**^**a**^
Pavord 2016 (INSPIRE, TRISTAN, SCO30002 trials) [99]	Global	–	–	–	–	–	–	–	–	–	–	–	–	**✔**^**c**^	–	–
Pikoula 2019 [84]	UK	–	**✔**	–	–	**✔**	–	–	–	–	–	–	**✔**	–	–	–
Roche 2017 (FLAME trial) [96]	Global	–	–	–	–	–	–	–	–	–	–	–	–	**✔**^**c**^	–	–
Rothnie 2018 [12]	UK	–	–	–	–	–	–	**✔**	–	–	–	–	–	–	–	–
Santibáñez 2016 [26]	Spain	**✔**	**✔**	–	–	**✔**	–	**✔**	✔	–	–	–	**✔**	–	–	**✔**^**b**^
Schuler 2018 (RIMTCORE study) [51]	Germany	**✔**	✔	–	**✔**	✔^**c**^	–	**✔**	✔	✔	**✔**	–	**✔**	–	–	**✔**^**a**^
Singh 2016 (TRILOGY trial) [52]	Global	**✔**	**✔**	–	–	**✔**	–	**✔**	–	–	–	–	**✔**	**✔**	–	**✔**
Søgaard 2016 [53]	Denmark	–	–	–	–	–	–	**✔**^**c**^	–	–	–	–	✔	–	–	–
Song 2018 [75]	South Korea	–	–	–	–	–	–	–	–	–	–	–	–	–	–	**✔**^**b**^
Stanford 2018 (HO-11–732 study) [54]	US	**✔**	–	–	–	–	–	**✔**	–	–	–	–	**✔**	–	–	–
Stanford 2019 [55]	US	–	–	–	–	–	–	**✔**	–	–	–	–	–	–	–	–
Sundh 2015 [76]	Sweden	–	–	–	–	✔	–	–	–	–	–	–	**✔**	–	–	**✔**^**a**^
Tsiligianni 2016 (UNLOCK study) [17]	Netherlands	–	–	–	–	–	–	**✔**	–	–	**✔**	–	–	–	–	–
Urwyler 2019 [77]	Switzerland	✔	✔	–	**✔**	✔	–	–	✔	–	–	–	**✔**	–	–	**✔**
Vedel-Krogh 2016 (Copenhagen General Population Study) [8]	Denmark	–	–	–	–	**✔**	–	**✔** [11]	–	–	–	–	–	**✔**	**✔**	**✔**
Vestbo 2017 (TRINITY trial) [56]	Global	**✔**	**✔**	–	–	**✔**	–	**✔**	–	–	–	–	**✔**	**✔**	–	**✔**
Vestbo 2019 [97]	Global	–	–	–	–	–	–	–	–	–	–	–	–	✔	–	–
Vogelmeier 2019 [60]	US & UK	–	–	–	–	–	–	✔	–	–	–	–	–	✔	–	–
Wallace 2019 [78]	US	–	–	–	–	–	–	–	–	–	–	–	–	–	–	**✔**^**b**^
Watz 2016 (WISDOM trial) [98]	US	–	–	–	–	–	–	–	–	–	–	–	–	**✔**^**c**^	–	–
Wei 2017 (TOLD study) [85]	Taiwan	✔	✔	✔	**✔**	✔	–	–	**✔**	–	–	–	✔	–	–	–
Wei 2018 [57]	China	–	–	–	–	–	–	**✔**	–	–	–	–	–	–	–	–
Westerik 2017 [7]	Netherlands	–	–	–	–	–	–	–	–	–	–	–	**✔**	–	–	–
Whalley 2019 (The Salford Lung study) [58]	UK	–	✔	–	–	✔	–	**✔**	–	–	–	–	✔	–	–	–
Wu 2018 [111]	China	–	–	–	–	✔	–	–	**✔**	–	–	–	–	–	–	–
Yii 2019 [21]	Singapore	✔	✔	**✔**	**✔**	✔	–	**✔**	✔	–	–	–	**✔**	–	–	–
Yohannes 2017 (ECLIPSE study) [16]	Global	**✔**	**✔**	–	**✔**	**✔**	–	**✔**	**✔**[25]	–	**✔**	–	**✔** [25, 30]	–	**✔**	**✔**
Yun 2018 (ECLIPSE + COPDGene study) [14]	Global	**✔**	**✔**	–	–	**✔**	–	**✔**	–	–	**✔**	**✔**	**✔**	✔	**✔**	**✔**
Zeiger 2018 [59]	US	–	**✔**	–	–	✔	–	**✔**	–	–	–	**✔**	**✔**	✔	–	**✔**

Bold cells denote studies reporting significant results. Results for each study were captured from the primary publications, except for any additional data captured from linked publications, which are cited in the table under the relevant prognostic factor

*6MWD* 6-min walk distance, *BDR* bronchodilator reversibility, *BMI* body mass index, *CAT* COPD Assessment Test, *COPD* chronic obstructive pulmonary disease, *mMRC* modified Medical Research Council, *QoL* quality of life

^a^Only forced expiratory volume in 1 s; ^b^Only disease severity; ^c^Association of two variables with exacerbations

**Fig. 4:**
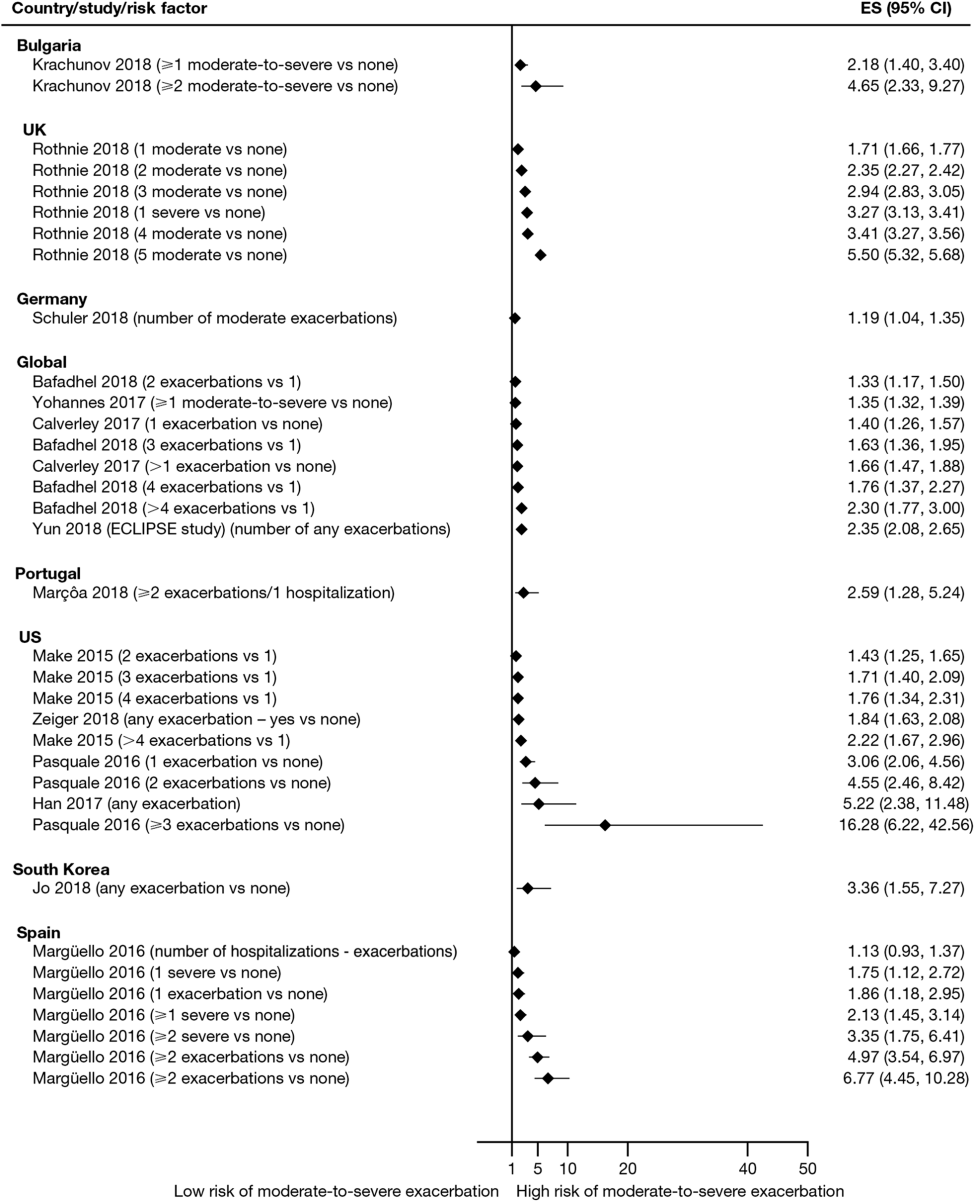
Exacerbation history as a risk factor for moderate-to-severe exacerbations. Yun 2018 included two studies; the study from which data were extracted (COPDGene or ECLIPSE) is listed in parentheses. *CI* confidence interval, *ES* effect size

**Fig. 5:**
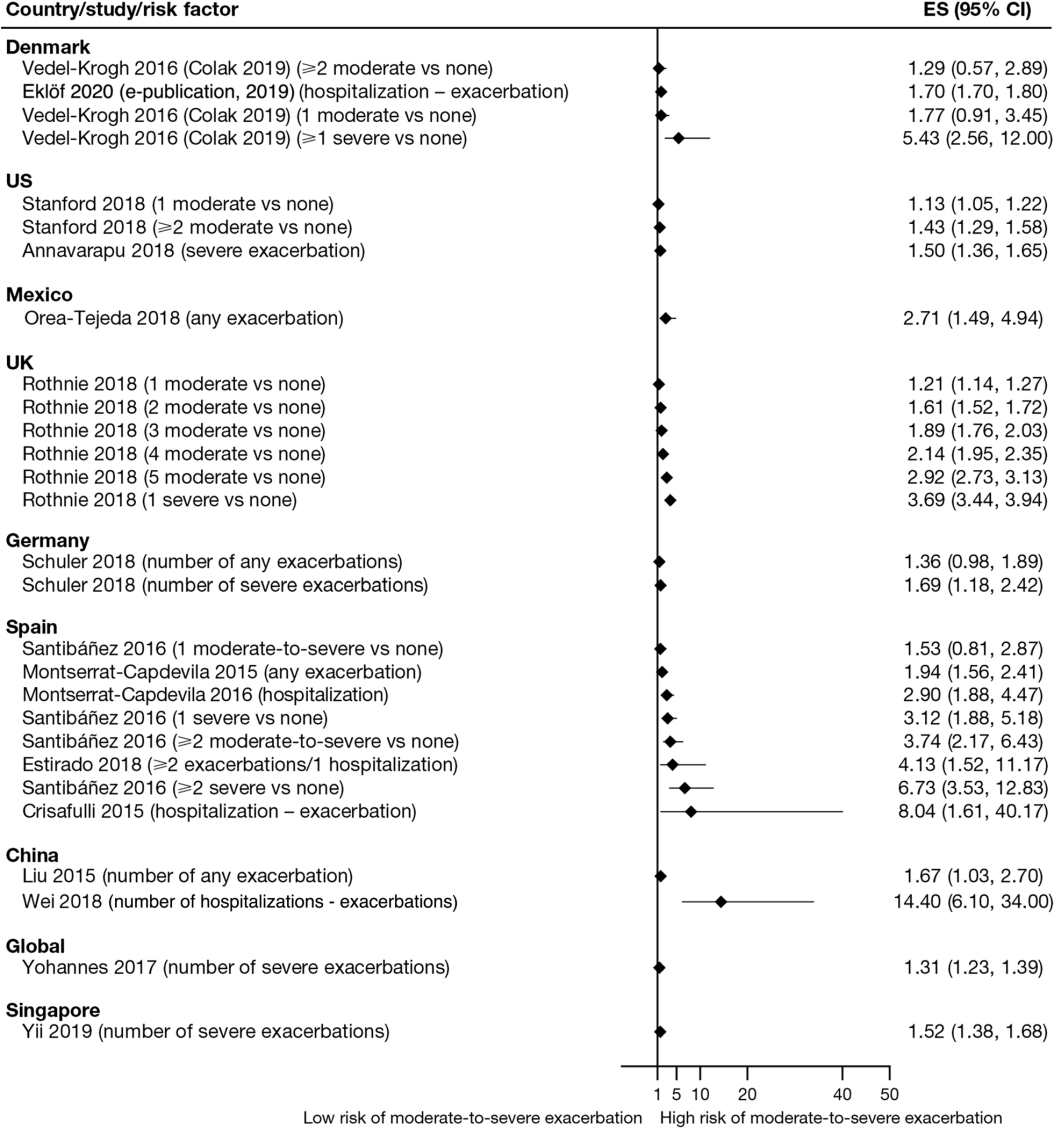
Exacerbation history as a risk factor for severe exacerbations. Where data have been extracted from a linked publication rather than the primary publication, the linked publication is listed in parentheses. *CI* confidence interval, *ES*, effect size

Exacerbation history within the past 12 months was the strongest predictor of future exacerbations. Across the studies assessing this predictor, 34 out of 35 studies (97.1%) reported a significant association between history of exacerbations and risk of future moderate-to-severe exacerbations (Table 3). Specifically, two or more exacerbations in the previous year or at least one hospitalization for COPD in the previous year were identified as reliable predictors of future moderate or severe exacerbations. Even one moderate exacerbation increased the risk of a future exacerbation, with the risk increasing further with each subsequent exacerbation (Fig. 4). A severe exacerbation was also found to increase the risk of subsequent exacerbation and hospitalization (Fig. 5). Patients experiencing one or more severe exacerbations were more likely to experience further severe exacerbations than moderate exacerbations [11, 12]. In contrast, patients with a history of one or more moderate exacerbations were more likely to experience further moderate exacerbations than severe exacerbations [11, 12].

**Fig. 6:**
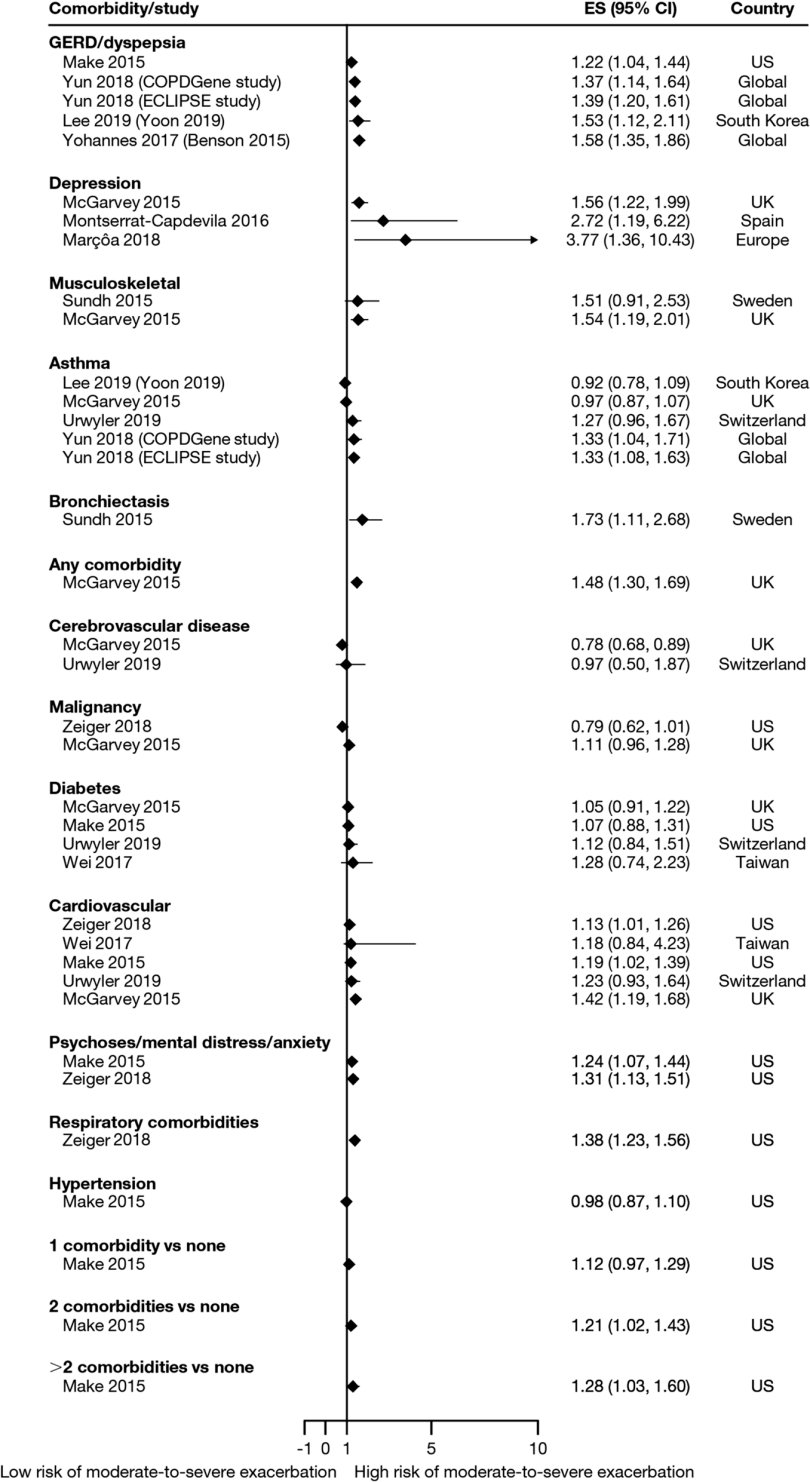
Comorbidities as risk factors for moderate-to-severe exacerbations. Yun 2018 included two studies; the study from which data were extracted (COPDGene or ECLIPSE) is listed in parentheses. Where data have been extracted from a linked publication rather than the primary publication, the linked publication is listed in parentheses. *CI* confidence interval, *ES* effect size, *GERD* gastroesophageal disease

**Fig. 7:**
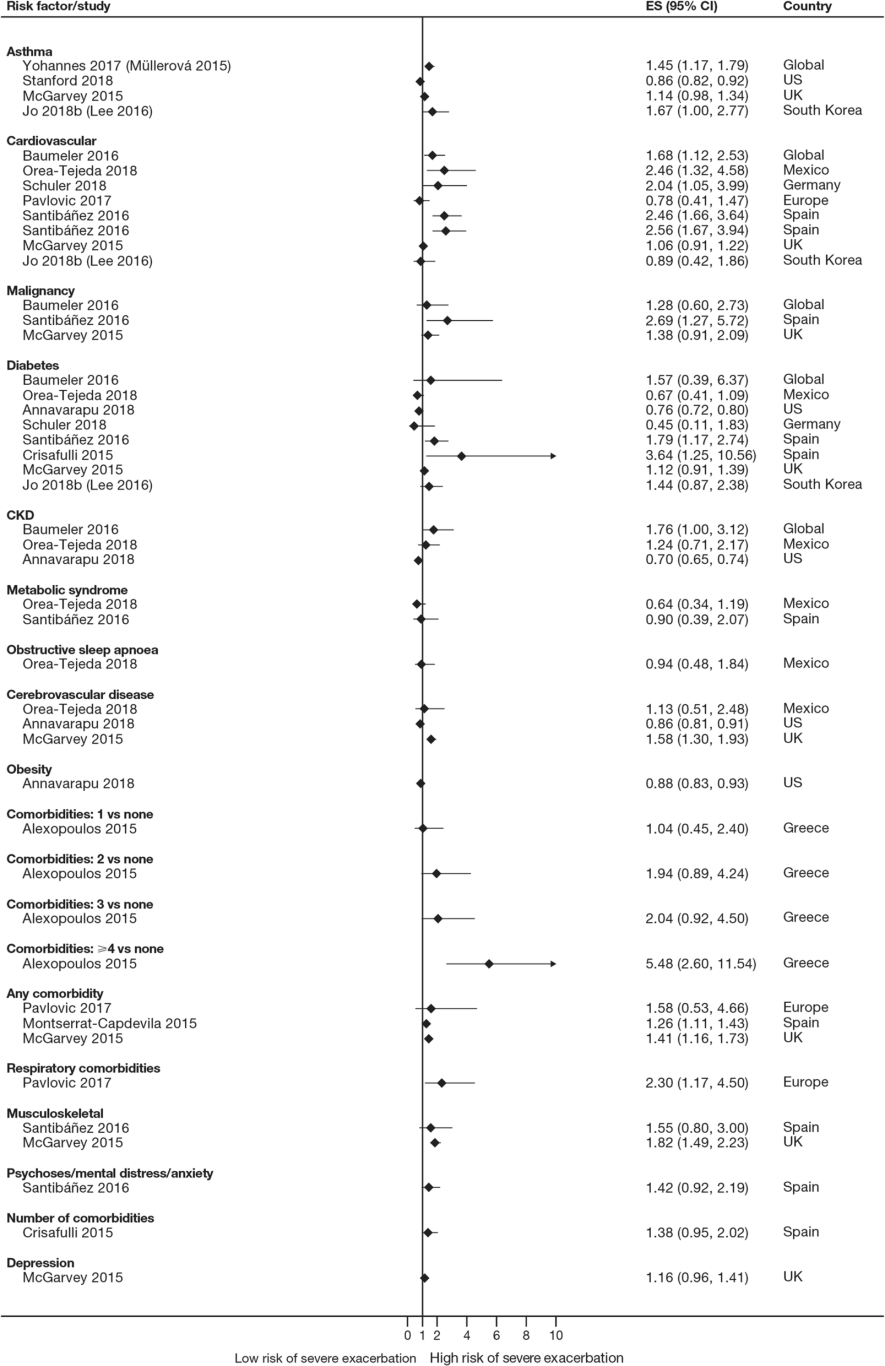
Comorbidities as risk factors for severe exacerbations. Where data have been extracted from a linked publication rather than the primary publication, the linked publication is listed in parentheses. *CI* confidence interval, *CKD*, chronic kidney disease, *ES* effect size

Overall, 35 studies assessed the association of comorbidities with the risk of exacerbation. All studies except one (97.1%) reported a positive association between comorbidities and the occurrence of moderate-to-severe exacerbations (Table 3). In addition to the presence of any comorbidity, specific comorbidities that were found to significantly increase the risk of moderate-to-severe exacerbations included anxiety and depression, cardiovascular comorbidities, gastroesophageal reflux disease/dyspepsia, and respiratory comorbidities (Fig. 6). Comorbidities that were significant risk factors for severe exacerbations included cardiovascular, musculoskeletal, and respiratory comorbidities, diabetes, and malignancy (Fig. 7). Overall, the strongest association between comorbidities and COPD readmissions in the emergency department was with cardiovascular disease. The degree of risk for both moderate-to-severe and severe exacerbations also increased with the number of comorbidities. A Dutch cohort study found that 88% of patients with COPD had at least one comorbidity, with hypertension (35%) and coronary heart disease (19%) being the most prevalent. In this cohort, the comorbidities with the greatest risk of frequent exacerbations were pulmonary cancer (odds ratio [OR] 1.85) and heart failure (OR 1.72) [7].

**Fig. 8:**
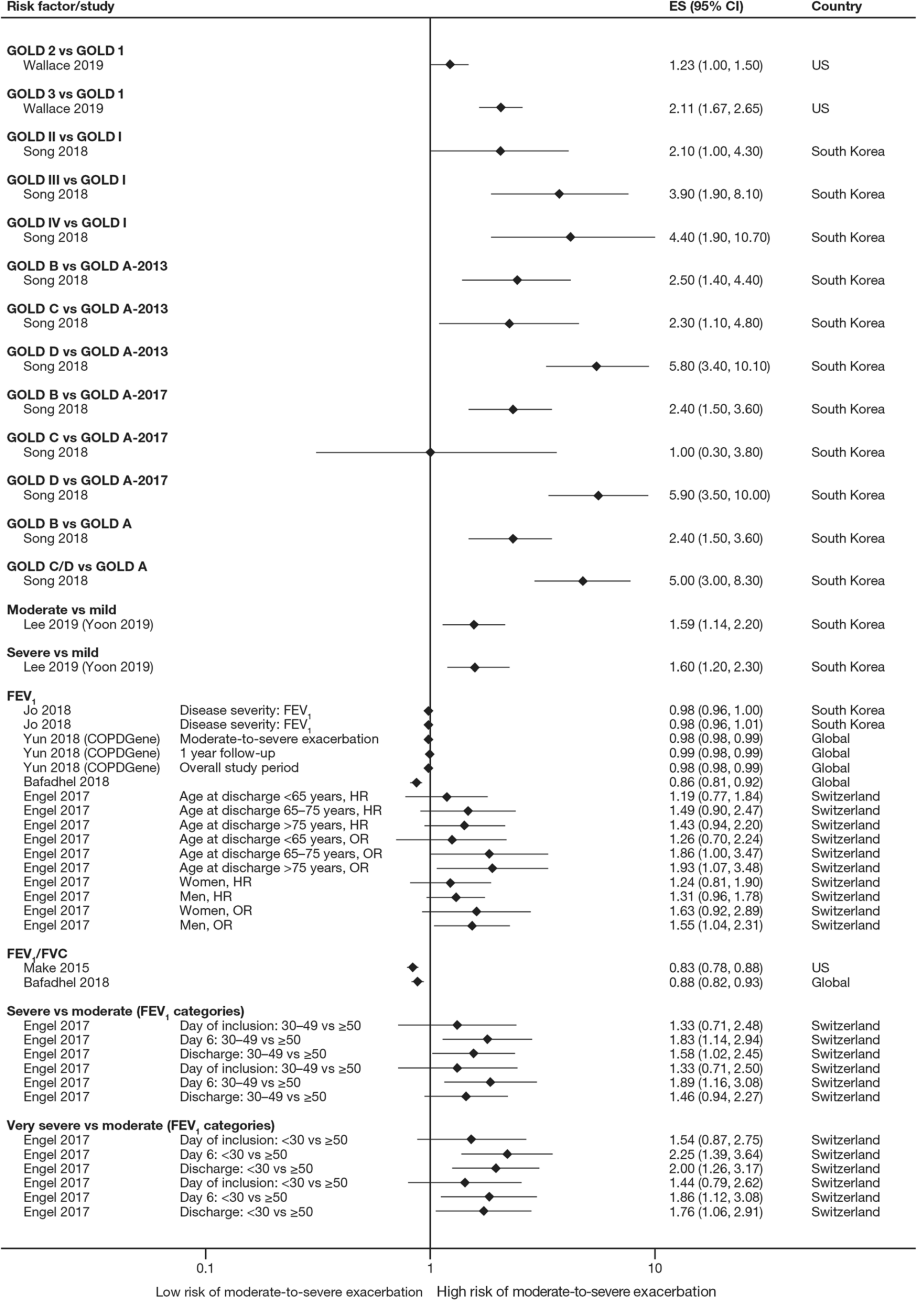
Disease severity as a risk factor for moderate-to-severe exacerbations. Yun 2018 included two studies; the study from which data were extracted (COPDGene or ECLIPSE) is listed in parentheses. Where data have been extracted from a linked publication rather than the primary publication, the linked publication is listed in parentheses. *CI* confidence interval, *ES* effect size, *FEV*_*1*_* f*orced expiratory volume in 1 s, *FVC*, forced vital capacity, *GOLD* Global Initiative for Obstructive Lung Disease, *HR* hazard ratio, *OR* odds ratio

**Fig. 9:**
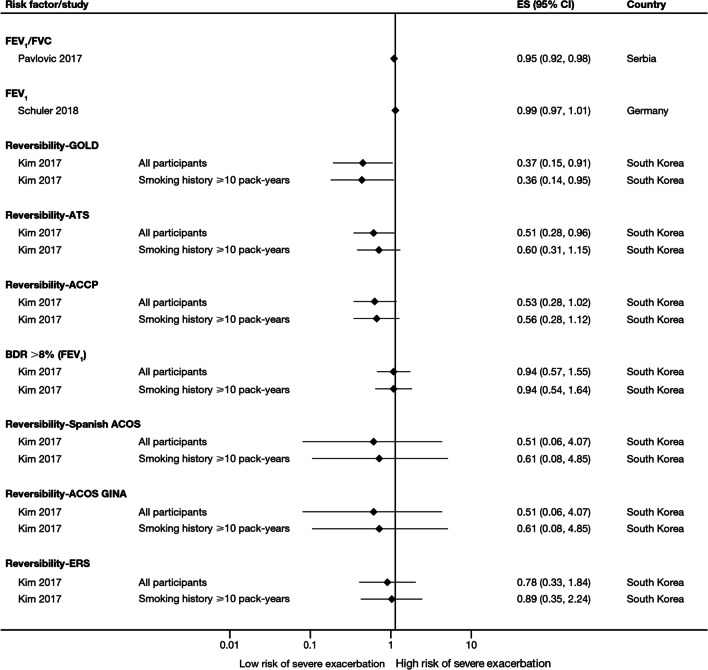
Disease severity and BDR as risk factors for severe exacerbations. *ACCP* American College of Chest Physicians, *ACOS* Asthma-COPD overlap syndrome, *ATS* American Thoracic Society, *BDR* bronchodilator reversibility, *CI* confidence interval, *ERS* European Respiratory Society, *ES* effect size, *FEV*_*1*_ forced expiratory volume in 1 s, *FVC* forced vital capacity, *GINA* Global Initiative for Asthma, *GOLD* Global Initiative for Obstructive Lung Disease

The majority of studies assessing disease severity or bronchodilator reversibility (39/41; 95.1%) indicated a significant positive relation between risk of future exacerbations and greater disease severity, as assessed by greater lung function impairment (in terms of lower FEV_1_, FEV_1_/forced vital capacity ratio, or forced expiratory flow [25–75]/forced vital capacity ratio) or more severe Global Initiative for Chronic Obstructive Lung Disease (GOLD) class A − D, and a positive relationship between risk of future exacerbations and lack of bronchodilator reversibility (Table 3, Figs. 8 and 9).

Of 21 studies assessing the relationship between blood eosinophil count and exacerbations (Table 3), 16 reported estimates for the risk of moderate or severe exacerbations by eosinophil count. A positive association was observed between higher eosinophil count and a higher risk of moderate or severe exacerbations, particularly in patients not treated with an inhaled corticosteroid (ICS); however, five studies reported a significant positive association irrespective of intervention effects. The risk of moderate-to-severe exacerbations was observed to be positively associated with various definitions of higher eosinophil levels (absolute counts: ≥ 200, ≥ 300, ≥ 340, ≥ 400, and ≥ 500 cells/mm^3^; % of blood eosinophil count: ≥ 2%, ≥ 3%, ≥ 4%, and ≥ 5%). Of note, one study found reduced efficacy of ICS in lowering moderate-to-severe exacerbation rates for current smokers versus former smokers at all eosinophil levels [13].

Of 12 studies assessing QoL scales, 11 (91.7%) studies reported a significant association between the worsening of QoL scores and the risk of future exacerbations (Table 3). Baseline SGRQ [14, 15], Center for Epidemiologic Studies Depression Scale (for which increased scores may indicate impaired QoL) [16], and Clinical COPD Questionnaire [17, 18] scores were found to be associated with future risk of moderate and/or severe COPD exacerbations. For symptom scores, six out of eight studies assessing the association between moderate-to-severe or severe exacerbations with COPD Assessment Test (CAT) scores reported a significant and positive relationship. Furthermore, the risk of moderate-to-severe exacerbations was found to be significantly higher in patients with higher CAT scores (≥ 10) [15, 19–21], with one study demonstrating that a CAT score of 15 increased predictive ability for exacerbations compared with a score of 10 or more [18]. Among 15 studies that assessed the association of modified Medical Research Council (mMRC) scores with the risk of moderate-to-severe or severe exacerbation, 11 found that the risk of moderate-to-severe or severe exacerbations was significantly associated with higher mMRC scores (≥ 2) versus lower scores. Furthermore, morning and night symptoms (measured by Clinical COPD Questionnaire) were associated with poor health status and predicted future exacerbations [17].

Of 36 studies reporting the relationship between smoking status and moderate-to-severe or severe exacerbations, 22 studies (61.1%) reported a significant positive association (Table 3). Passive smoking was also significantly associated with an increased risk of severe exacerbations (OR 1.49) [20]. Of note, three studies reported a significantly lower rate of moderate-to-severe exacerbations in current smokers compared with former smokers [22–24].

A total of 14 studies assessed the association of body mass index (BMI) with the occurrence of frequent moderate-to-severe exacerbations in patients with COPD. Six out of 14 studies (42.9%) reported a significant negative association between exacerbations and BMI (Table 3). The risk of moderate and/or severe COPD exacerbations was highest among underweight patients compared with normal and overweight patients [23, 25–28].

In the 29 studies reporting an association between age and moderate or severe exacerbations, more than half found an association of older age with an increased risk of moderate-to-severe exacerbations (58.6%; Table 3). Four of these studies noted a significant increase in the risk of moderate-to-severe or severe exacerbations for every 10-year increase in age [25, 26, 29, 30]. However, 12 studies reported no significant association between age and moderate-to-severe or severe exacerbation risk.

Sixteen out of 33 studies investigating the impact of sex on exacerbation risk found a significant association (48.5%; Table 3). Among these, ten studies reported that female sex was associated with an increased risk of moderate-to-severe exacerbations, while six studies showed a higher exacerbation risk in males compared with females. There was some variation in findings by geographic location and exacerbation severity (Additional file 2: Figs. S1 and S2). Notably, when assessing the risk of severe exacerbations, more studies found an association with male sex compared with female sex (6/13 studies vs 1/13 studies, respectively).

Both studies evaluating associations between exacerbations and environmental factors reported that colder temperature and exposure to major air pollution (NO_2_, O_3_, CO, and/or particulate matter ≤ 10 μm in diameter) increased hospital admissions due to severe exacerbations and moderate-to-severe exacerbation rates [31, 32].

Four studies assessed the association of 6-min walk distance with the occurrence of frequent moderate-to-severe exacerbations (Table 3). One study (25.0%) found that shorter 6-min walk distance (representing low physical activity) was significantly associated with a shortened time to severe exacerbation, but the effect size was small (hazard ratio 0.99) [33].

Five out of six studies assessing the relationship between race or ethnicity and exacerbation risk reported significant associations (Table 3). Additionally, one study reported an association between geographic location in the US and exacerbations, with living in the Northeast region being the strongest predictor of severe COPD exacerbations versus living in the Midwest and South regions [34].

Overall, seven studies assessed the association of biomarkers with risk of future exacerbations (Table 3), with the majority identifying significant associations between inflammatory biomarkers and increased exacerbation risk, including higher C-reactive protein levels [8, 35], fibrinogen levels [8, 30], and white blood cell count [8, 15, 16].

## Discussion

This SLR has identified several demographic and clinical characteristics that predict the future risk of COPD exacerbations. Key factors associated with an increased risk of future moderate-to-severe exacerbations included a history of prior exacerbations, worse disease severity and bronchodilator reversibility, the presence of comorbidities, a higher eosinophil count, and older age (Fig. 2). These prognostic factors may help clinicians identify patients at high risk of exacerbations, which are a major driver of the burden of COPD, including morbidity and mortality [36].

Findings from this review summarize the existing evidence, validating the previously published literature [6, 9, 23] and suggesting that the best predictor of future exacerbations is a history of exacerbations in the prior year [8, 11–14, 16–23, 26, 29, 34, 35, 37–60]. In addition, the effect size generally increased with the number of prior exacerbations, with a stronger effect observed with prior severe versus moderate exacerbations. This effect was observed across regions, including in Europe and North America, and in several global studies. This relationship represents a vicious circle, whereby one exacerbation predisposes a patient to experience future exacerbations and leading to an ever-increasing disease burden, and emphasizes the importance of preventing the first exacerbation event through early, proactive exacerbation prevention. The finding that prior exacerbations tended to be associated with future exacerbations of the same severity suggests that the severity of the underlying disease may influence exacerbation severity. However, the validity of the traditional classification of exacerbation severity has recently been challenged [61], and further work is required to understand relationships with objective assessments of exacerbation severity.

In addition to exacerbation history, disease severity and bronchodilator reversibility were also strong predictors for future exacerbations [8, 14, 16, 18–20, 22–24, 26, 28, 29, 33, 37, 40, 43–46, 48, 50–52, 56, 59, 62–78]. The association with disease severity was noted in studies that used GOLD disease stages 1–4 and those that used FEV_1_ percent predicted and other lung function assessments as continuous variables. Again, this risk factor is self-perpetuating, as evidence shows that even a single moderate or severe exacerbation may almost double the rate of lung function decline [79]. Accordingly, disease severity and exacerbation history may be correlated. Margüello et al. concluded that the severity of COPD could be associated with a higher risk of exacerbations, but this effect was partly determined by the exacerbations suffered in the previous year [23]. It should be noted that FEV_1_ is not recommended by GOLD for use as a predictor of exacerbation risk or mortality alone due to insufficient precision when used at the individual patient level [5].

Another factor that should be considered when assessing individual exacerbation risk is the presence of comorbidities [7, 14, 16, 18–22, 24–28, 30, 33–35, 40, 41, 44–48, 51–54, 56, 58, 59, 63, 64, 73, 74, 76, 77, 80–85]. Comorbidities are common in COPD, in part due to common risk factors (e.g., age, smoking, lifestyle factors) that also increase the risk of other chronic diseases [7]. Significant associations were observed between exacerbation risk and comorbidities, such as anxiety and depression, cardiovascular disease, diabetes, and respiratory comorbidities. As with prior exacerbations, the strength of the association increased with the number of comorbidities. Some comorbidities that were found to be associated with COPD exacerbations share a common biological mechanism of systemic inflammation, such as cardiovascular disease, diabetes, and depression [86]. Furthermore, other respiratory comorbidities, including asthma and bronchiectasis, involve inflammation of the airways [87]. In these patients, optimal management of comorbidities may reduce the risk of future COPD exacerbations (and improve QoL), although further research is needed to confirm the efficacy of this approach to exacerbation prevention. As cardiovascular conditions, including hypertension and coronary heart disease, are the most common comorbidities in people with COPD [7], reducing cardiovascular risk may be a key goal in reducing the occurrence of exacerbations. For other comorbidities, the mechanism for the association with exacerbation risk may be related to non-biological factors. For example, in depression, it has been suggested that the mechanism may relate to greater sensitivity to symptom changes or more frequent physician visits [88].

There is now a growing body of evidence reporting the relationship between blood eosinophil count and exacerbation risk [8, 13, 14, 20, 37, 48, 52, 56, 59, 60, 62, 89–99]. Data from many large clinical trials (SUNSET [89], FLAME [96], WISDOM [98], IMPACT [13], TRISTAN [99], INSPIRE [99], KRONOS [91], TRIBUTE [48], TRILOGY [52], TRINITY [56]) have also shown relationships between treatment, eosinophil count, and exacerbation rates. Evidence shows that eosinophil count, along with other effect modifiers (e.g., exacerbation history), can be used to predict reductions in exacerbations with ICS treatment. Identifying patients most likely to respond to ICS should contribute to personalized medicine approaches to treat COPD. One challenge in drawing a strong conclusion from eosinophil counts is the choice of a cut-off value, with a variety of absolute and percentage values observed to be positively associated with the risk of moderate-to-severe exacerbations. The use of absolute counts may be more practical, as these are not affected by variations in other immune cell numbers; however, there is a lack of consensus on this point [100].

Across the studies examined, associations between sex and the risk of moderate and/or severe exacerbations were variable [14, 16, 18, 20–24, 26–29, 37, 40, 42, 44–48, 51, 52, 56, 58, 59, 63, 73, 74, 77, 80, 83–85]. A greater number of studies showed an increased risk of exacerbations in females compared with males. In contrast, some studies failed to detect a relationship, suggesting that country-specific or cultural factors may play a role. A majority of the included studies evaluated more male patients than female patients; to further elucidate the relationship between sex and exacerbations, more studies in female patients are warranted. Over half of the studies that assessed the relationship between age and exacerbation risk found an association between increasing age and increasing risk of moderate-to-severe COPD exacerbations [14, 16, 18, 20–24, 26–29, 33, 40, 42, 44, 45, 47, 51, 52, 54, 56, 63, 73, 74, 77, 80, 83, 85].

Our findings also suggested that patients with low BMI have greater risk of moderate and/or severe exacerbations. The mechanism underlying this increased risk in underweight patients is poorly understood; however, loss of lean body mass in patients with COPD may be related to ongoing systemic inflammation that impacts skeletal muscle mass [101–103].

A limitation of this SLR, that may have resulted in some studies with valid results being missed, was the exclusion of non-English-language studies and the limitation by date; however, the search strategy was otherwise broad, resulting in the review of a large number of studies. The majority of studies captured in this SLR were from Europe, North America, and Asia. The findings may therefore be less generalizable to patients in other regions, such as Africa or South America. Given that one study reported an association between geographic location within different regions of the US and exacerbations [34], it is plausible that risk of exacerbations may be impacted by global location. As no formal meta-analysis was planned, the assessments are based on a qualitative synthesis of studies. A majority of the included studies looked at exposures of certain factors (e.g., history of exacerbations) at baseline; however, some of these factors change over time, calling into question whether a more sophisticated statistical analysis should have been conducted in some cases to consider time-varying covariates. Our results can only inform on associations, not causation, and there are likely bidirectional relationships between many factors and exacerbation risk (e.g., health status). Finally, while our review of the literature captured a large number of prognostic factors, other variables such as genetic factors, lung microbiome composition, and changes in therapy over time have not been widely studied to date, but might also influence exacerbation frequency [104]. Further research is needed to assess the contribution of these factors to exacerbation risk.

This SLR captured publications up to July 2019. However, further studies have since been published that further support the prognostic factors identified here. For example, recent studies have reported an increased risk of exacerbations in patients with a history of exacerbations [105], comorbidities [106], poorer lung function (GOLD stage) [105], higher symptomatic burden [107], female sex [105], and lower BMI [106, 108].

## Conclusions

In summary, the literature assessing risk factors for moderate-to-severe COPD exacerbations shows that there are associations between several demographic and disease characteristics with COPD exacerbations, potentially allowing clinicians to identify patients most at risk of future exacerbations. Exacerbation history, comorbidities, and disease severity or bronchodilator reversibility were the factors most strongly associated with exacerbation risk, and should be considered in future research efforts to develop prognostic tools to estimate the likelihood of exacerbation occurrence. Importantly, many prognostic factors for exacerbations, such as symptom burden, QoL, and comorbidities, are modifiable with optimal pharmacologic and non-pharmacologic treatments or lifestyle modifications. Overall, the evidence suggests that, taken together, predicting and reducing exacerbation risk is an achievable goal in COPD.

## Supplementary Information

**Additional file1: Table S1.** Search strategies.** Table S2.** List of included studies with linked publications. **Table S3.** Study characteristics across the 76 included studies. **Table S4. **Clinical characteristics of the patients assessed across the included studies.

**Additional file 2: Fig. S1. **Sex (male vs female) as a risk factor for moderate-to-severe exacerbations. **Fig. S2.** Sex (male vs female) as a risk factor for severe exacerbations.

## Data Availability

The datasets used and/or analyzed during the current study are available from the corresponding author on reasonable request.

## Abbreviations

BMI: Body mass index
CAT: COPD Assessment Test
COPD: Chronic obstructive pulmonary disease
FEV_1_: Forced expiratory volume in 1 s
GOLD: Global Initiative for Chronic Obstructive Lung Disease
ICS: Inhaled corticosteroid
mMRC: Modified Medical Research Council
OR: Odds ratio
QoL: Quality of life
SGRQ: St. George’s Respiratory Questionnaire
SLR: Systematic literature review
