# Supplementary material for: Prognostic risk factors for moderate-to-severe exacerbations in patients with chronic obstructive pulmonary disease: a systematic literature review
Source: Respir Res. 2022 Aug 23;23:213. doi: 10.1186/s12931-022-02123-5 (PMC9396841; doi:10.1186/s12931-022-02123-5)
Supplement: Supplementary file 1 — Additional file1: Table S1. Search strategies. Table S2. List of included studies with linked publications. Table S3. Study characteristics across the 76 included studies. Table S4. Clinical characteristics of the patients assessed across the included studies. [file 12931_2022_2123_MOESM1_ESM.docx]

Additional File 1

Supplementary Table 1 Search strategies

| No. | Query/search history | Search facet |
| --- | --- | --- |
| Embase and MEDLINE (searched through Embase.com on 15 July 2019) | | |
| #1 | ‘chronic obstructive lung disease'/exp OR ‘chronic obstructive lung disease'/syn OR ‘chronic airflow obstruction’ OR ‘chronic airway obstruction’ OR ‘chronic obstructive bronchitis’ OR ‘chronic obstructive bronchopulmonary disease’ OR ‘chronic obstructive lung disorder’ OR ‘chronic obstructive pulmonary disease’ OR ‘chronic obstructive pulmonary disorder’ OR ‘chronic obstructive respiratory disease’ OR ‘lung chronic obstructive disease’ OR ‘lung disease, chronic obstructive’ OR ‘lung diseases, obstructive’ OR ‘obstructive lung disease’ OR ‘obstructive lung disease, chronic’ OR ‘obstructive pulmonary disease’ OR ‘obstructive respiratory disease’ OR ‘obstructive respiratory tract disease’ OR ‘pulmonary disease, chronic obstructive’ OR ‘pulmonary disorder, chronic obstructive’ OR ‘chronic obstructive lung disease':ab,ti OR copd:ab,ti OR ‘chronic obstructive pulmonary disease’ | 149,218 |
| #2 | 'exacerbation':ab,ti OR exacerbati*:ab,ti | 85,360 |
| #3 | #1 AND #2 | 20,584 |
| #4 | 'predictors':ab,ti OR predict*:ab,ti OR progn*:ab,ti OR 'risk factor*':ab,ti | 3,147,764 |
| #5 | #3 AND #4 | 6090 |
| #6 | #5 AND [2009-2019]/py | 5349 |
| #7 | #6 AND [2009-2019]/py AND [english]/lim | 5151 |
| #8 | #7 AND ([conference review]/lim OR [editorial]/lim OR [letter]/lim OR [note]/lim OR [review]/lim OR [short survey]/lim) | 352 |
| #9 | #7 AND [animals]/lim NOT ([humans]/lim AND [animals]/lim) | 107 |
| #10 | #8 OR #9 | 457 |
| #11 | #7 NOT #10 | 4694 |
| MEDLINE In-Process (searched through Pubmed.com interface on 16 July 2019) | | |
| #1 | Search “chronic obstructive lung disease” | 3928 |
| #2 | Search (“chronic bronchitis” OR emphysema) | 44,044 |
| #3 | Search (chronic*) AND bronchiti* | 16,179 |
| #4 | Search (obstruct*[Title/Abstract]) AND (pulmonary OR lung* OR airway* OR airflow* OR bronch* OR respirat*[Title/Abstract]) | 111,538 |
| #5 | Search #1 OR #2 OR #3 OR #4 | 151,734 |
| #6 | Search “exacerbation” | 33,321 |
| #7 | Search (exacerbation [Title/Abstract]) OR (exacerbati*[Title/Abstract]) | 52,360 |
| #8 | #6 OR #7 | 52,363 |
| #9 | #5 AND #9 | 11,174 |
| #10 | Search (predictors [Title/Abstract]) OR (predict*[Title/Abstract]) OR (prognostic [Title/Abstract]) OR (progn*[Title/Abstract]) OR (risk factor* [Title/Abstract]) | 2,304,002 |
| #11 | #9 AND #10 | 2796 |
| #12 | (#11 AND (inprocess[sb] OR pubstatusaheadofprint)) | 199 |
| Cochrane (searched through Cochrane library interface on 16 July 2019) | | |
| #1 | MeSH descriptor: [Pulmonary Disease, Chronic Obstructive] explode all trees | 4813 |
| #2 | MeSH descriptor: [Bronchitis, Chronic] explode all trees | 146 |
| #3 | MeSH descriptor: [Emphysema] explode all trees | 195 |
| #4 | (chronic* near/3 bronchiti*):ti,ab,kw | 1957 |
| #5 | (emphysema* or copd or coad):ti,ab,kw | 15,759 |
| #6 | (obstruct* near/3 (pulmonary or lung* or airway* or airflow* or bronch* or respirat*)):ti,ab,kw | 17,913 |
| #7 | #1 or #2 or #3 or #4 or #5 or #6 | 24,647 |
| #8 | ("exacerbation" OR exacerbati*):ti,ab,kw | 14,833 |
| #9 | ("predictors" OR predict* OR prognostic OR progn* OR "risk factor*"):ti,ab,kw | 144,761 |
| #10 | #7 AND #8 AND #9 | 1141 |
| #11 | #10 NOT (embase or pubmed):an | 287 |
| #12 | #11 (with Publication Year from 2009 to 2019, in Trials) | 187 |

Supplementary Table 2 List of included studies with linked publications

| Primary publication | Study name | Linked publications with relevant data | Linked publications that met review criteria but did not provide any additional information beyond what was available from primary study |
| --- | --- | --- | --- |
| Adir 2018 [1] | NA | NA | NA |
| Alexopoulos 2015 [2] | GOLDEN study | NA | NA |
| Annavarapu 2018 [3] | NA | NA | NA |
| Bade 2019 [4] | NA | NA | NA |
| Bafadhel 2018 [5] | NA | NA | NA |
| Bartels 2018 [6] | NA | NA | Van Eeden 2018 [7] |
| Baumeler 2016 [8] | PROMISE-COPD study | NA | Egli 2018 [9], Mandal 2015 [10], Papakonstantinou 2019 [11], Mandal 2016 [12], Papakonstantinou 2018 [13] |
| Calverley 2018 [14] | TIOSPIR study | Calverley 2017 [15] | McGarvey 2016 [16] |
| Chapman 2018 [17] | SUNSET trial | NA | NA |
| Couillard 2017 [18] | NA | NA | NA |
| Crisafulli 2015 [19] | NA | NA | NA |
| de Miguel-Díez 2019 [20] | NA | NA | NA |
| Eklöf 2020  (e-publication, 2019) [21] | NA | NA | NA |
| Emura 2019 [22] | NA | NA | NA |
| Engel 2017 [23] | REDUCE study | NA | NA |
| Erol 2018 [24] | NA | NA | NA |
| Estirado 2018 [25] | NA | NA | NA |
| Ferguson 2018 [26] | KRONOS study | NA | NA |
| Fuhrman 2017 [27] | NA | NA | NA |
| Han 2017 [28] | SPIROMICS study | Diamond 2017 [29], Putcha 2015 [30] | Keene 2017 [31], Paul 2017 [32] |
| Han 2018 [33] | NA | NA | NA |
| Huang 2018 [34] | NA | NA | NA |
| Jo 2018 [35] | NA | NA | NA |
| Jo 2018b [36] | NA | Lee 2016 [37] | NA |
| Jung 2015 [38] | NA | NA | Lee 2015 [39] |
| Kim 2017 [40] | NA | NA | Kim 2017 [41] |
| Kim 2019 [42] | COPDGene study | Iyer 2017 [43], Abston 2017 [44] | Bodduluri 2018 [45], Kim 2017 [46] |
| Ko 2019 [47] | NA | NA | NA |
| Kobayashi 2018 [48] | NA | NA | NA |
| Krachunov 2017 [49] | NA | NA | NA |
| Krachunov 2018 [50] | NA | NA | NA |
| Lau 2017 [51] | NA | NA | NA |
| Lee 2019 [52] | NA | Yoon 2019 [53] | NA |
| Liu 2015 [54] | NA | NA | NA |
| MacDonald 2019 [55] | NA | NA | NA |
| Make 2015 [56] | NA | NA | NA |
| Marçôa 2018 [57] | NA | NA | NA |
| Margüello 2016 [58] | NA | NA | NA |
| McGarvey 2015 [59] | NA | NA | NA |
| Montserrat-Capdevila 2015 [60] | NA | NA | NA |
| Montserrat-Capdevila 2016 [61] | NA | NA | NA |
| Müllerová 2019 [62] | HO-17-18395 study | NA | NA |
| Orea-Tejeda 2018 [63] | NA | NA | NA |
| Papi 2018 [64] | TRIBUTE trial | NA | NA |
| Pascoe 2019 [65] | IMPACT trial | Lipson 2018 [66] | NA |
| Pasquale 2016 [67] | NA | NA | NA |
| Pavlovic 2017 [68] | NA | NA | NA |
| Pavord 2016 [69] | INSPIRE, TRISTAN, SCO30002 trial | NA | NA |
| Pikoula 2019 [70] | NA | NA | NA |
| Roche 2017 [71] | FLAME trial | NA | NA |
| Rothnie 2018 [72] | NA | NA | NA |
| Santibáñez 2016 [73] | NA | NA | Santibáñez 2015 [74] |
| Schuler 2018 [75] | RIMTCORE study | NA | NA |
| Singh 2016 [76] | TRILOGY trial | NA | NA |
| Søgaard 2016 [77] | NA | NA | NA |
| Song 2018 [78] | NA | NA | NA |
| Stanford 2018 [79] | HO-11-732 study | NA | Stanford 2016 [80] |
| Stanford 2019 [81] | NA | NA | NA |
| Sundh 2015 [82] | NA | NA | NA |
| Tsiligianni 2016 [83] | UNLOCK study | NA | NA |
| Urwyler 2019 [84] | NA | NA | NA |
| Vedel-Krogh 2016 [85] | Copenhagen General Population Study | Colak 2019 [86] | Vedel-Krogh 2015 [87], Ingebrigtsen 2015 [88] |
| Vestbo 2017 [89] | TRINITY trial | NA | NA |
| Vestbo 2019 [90] | NA | NA | NA |
| Vogelmeier 2019 [91] | NA | NA | NA |
| Wallace 2019 [92] | NA | NA | NA |
| Watz 2016 [93] | WISDOM trial | Sajkov 2017 [94] | Calverley 2017 [95], Frith 2019 [96], Watz 2018 [97], Wouters 2017 [98], Calverley 2017 [99] |
| Wei 2017 [100] | TOLD study | NA | NA |
| Wei 2018 [101] | NA | NA | NA |
| Westerik 2017 [102] | NA | NA | NA |
| Whalley 2019 [103] | The Salford Lung Study | NA | NA |
| Wu 2018 [104] | NA | NA | NA |
| Yii 2019 [105] | NA | NA | NA |
| Yohannes 2017 [106] | ECLIPSE study | Wilke 2015 [107], Müllerová 2015 [108], Benson 2015 [109] | Benson 2015 [110] |
| Yun 2018 [111] | ECLIPSE + COPDGene study | NA | NA |
| Zeiger 2018 [112] | NA | NA | NA |

*NA* not applicable

Supplementary Table 3 Study characteristics across the 76 included studies

| Study | Study design | Country | Setting | Sample size | Exacerbation severity |
| --- | --- | --- | --- | --- | --- |
| Adir 2018 [1] | Retrospective obs. | Israel | Single center | 992 | Severe |
| Alexopoulos 2015 (GOLDEN study) [2] | Cross-sectional | Greece | Multicenter | 6125 | Severe |
| Annavarapu 2018 [3] | Retrospective obs. | US | Multicenter | 45,722 | Severe |
| Bade 2019 [4] | Retrospective obs. | US | Multicenter | 48,888 | Severe |
| Bafadhel 2018 [5] | RCT | Global | Multicenter international | 4528 | Moderate-to-severe |
| Bartels 2018 [6] | Retrospective obs. | Canada | Single center | 511 | Severe |
| Baumeler 2016 (PROMISE-COPD study) [8] | Prospective obs. | European countries (8) | Multicenter international | 638 | Severe |
| Bodduluri 2018 (COPDGene study) [45] | Cross-sectional | US | Multicenter | 8135 | Severe |
| Calverley 2018 (TIOSPIR study) [14] | RCT | Global | Multicenter international | 17,135 | Moderate-to-severe |
| Chapman 2018 (SUNSET trial) [17] | RCT | Global | Multicenter international | 1053 | Moderate-to-severe |
| Couillard 2017 [18] | Retrospective obs. | Canada | Multicenter | 167 | Severe |
| Crisafulli 2015 [19] | Prospective obs. | Spain | Multicenter | 125 | Severe |
| de Miguel-Díez 2019 [20] | Retrospective obs. | Spain | Multicenter | 162,338 | Severe |
| Eklöf 2020  (e-publication, 2019) [21] | Retrospective obs. | Denmark | Multicenter | 22,053 | Severe |
| Emura 2019 [22] | Retrospective obs. | Japan | Single center | 432 | Severe |
| Engel 2017 (REDUCE study) [23] | RCT | Switzerland | Multicenter | 311 | Moderate-to-severe |
| Erol 2018 [24] | Retrospective obs. | Turkey | Single center | 225 | Moderate-to-severe |
| Estirado 2018 [25] | Prospective obs. | Spain | Single center | 195 | Severe |
| Ferguson 2018 (KRONOS study) [26] | RCT | Global | Multicenter international | 1902 | Moderate-to-severe |
| Fuhrman 2017 [27] | Retrospective obs. | France | Multicenter | 58,144 | Severe |
| Han 2017 (SPIROMICS study) [28] | Prospective obs. | US | Multicenter | 1105 | Moderate-to-severe |
| Han 2018 [33] | Retrospective obs. | Taiwan | Single center | 628 | Moderate-to-severe |
| Huang 2018 [34] | Retrospective obs. | Taiwan | Single center | 296 | Severe |
| Jo 2018 [35] | Prospective obs. | South Korea | Multicenter | 246 | Moderate-to-severe |
| Jo 2018b [36] | Retrospective obs. | South Korea | Multicenter | 1320 | Moderate-to-severe |
| Jung 2015 [38] | Prospective obs. | South Korea | Single center | 118 | Moderate-to-severe |
| Kim 2017 [40] | Prospective obs. | South Korea | Multicenter | 854 | Severe |
| Ko 2019 [47] | Prospective obs. | China | Single center | 346 | Severe |
| Kobayashi 2018 [48] | Prospective obs. | Japan | Multicenter | 401 | Severe |
| Krachunov 2017 [49] | Prospective obs. | Bulgaria | Single center | 249 | Moderate-to-severe |
| Krachunov 2018 [50] | Prospective obs. | Bulgaria | Single center | 465 | Moderate-to-severe |
| Lau 2017 [51] | Retrospective obs. | US | Multicenter | 339,389 | Severe |
| Lee 2019 [52] | Retrospective and prospective obs. | South Korea | Multicenter international | 1144 | Moderate-to-severe |
| Liu 2015 [54] | Prospective obs. | China | Single center | 176 | Very severe |
| MacDonald 2019 [55] | Retrospective and prospective obs. | Australia | Single center | 341 | Severe |
| Make 2015 [56] | RCT | US | Multicenter | 3141 | Moderate-to-severe |
| Marçôa 2018 [57] | Prospective obs. | Portugal | Multicenter | 200 | Moderate-to-severe |
| Margüello 2016 [58] | Retrospective obs. | Spain | Multicenter | 900 | Moderate-to-severe |
| McGarvey 2015 [59] | Retrospective obs. | UK | Multicenter | 9219 | Moderate-to-severe |
| Montserrat-Capdevila 2015 [60] | Retrospective obs. | Spain | Multicenter | 2501 | Severe |
| Montserrat-Capdevila 2016 [61] | Prospective obs. | Spain | Single center | 512 | Moderate-to-severe |
| Müllerová 2019 (HO-17-18395 study) [62] | Retrospective obs. | US | Multicenter | 34,268 | Moderate-to-severe |
| Orea-Tejeda 2018 [63] | Prospective obs. | Mexico | Single center | 133 | Severe |
| Papi 2018 (TRIBUTE trial) [64] | RCT | Global | Multicenter international | 1532 | Moderate-to-severe |
| Pascoe 2019 (IMPACT trial) [65] | RCT | Global | Multicenter international | 10,333 | Moderate-to-severe |
| Pasquale 2016 [67] | Retrospective obs. | US | Multicenter | 1159 | Moderate-to-severe |
| Pavlovic 2017 [68] | Case-control | Serbia | Single center | 512 | Severe |
| Pavord 2016 (INSPIRE, TRISTAN, SCO30002 trials) [69] | RCT | Global | Multicenter international | 3045 [1269 (INSPIRE), 1403 (TRISTAN), and 373 (SCO30002)] | Moderate-to-severe |
| Pikoula 2019 [70] | Retrospective obs. | UK | Multicenter | 30,961 | Severe |
| Roche 2017 (FLAME trial) [71] | RCT | Global | Multicenter international | 3349 | Moderate-to-severe |
| Rothnie 2018 [72] | Retrospective obs. | UK | Multicenter | 99,574 | Moderate-to-severe |
| Santibáñez 2016 [73] | Retrospective obs. | Spain | Single center | 900 | Moderate-to-severe |
| Schuler 2018 (RIMTCORE study) [75] | RCT | Germany | Single center | 383 | Moderate-to-severe |
| Singh 2016 (TRILOGY trial) [76] | RCT | Global | Multicenter international | 1368 | Moderate-to-severe |
| Søgaard 2016 [77] | Retrospective obs. | Denmark | Multicenter | 52,520 | Severe |
| Song 2018 [78] | Prospective obs. | South Korea | Multicenter | 1880 | Moderate-to-severe |
| Stanford 2018 (HO-11-732 study) [79] | Retrospective obs. | US | Multicenter | 223,824 | Severe |
| Stanford 2019 [81] | Retrospective obs. | US | Multicenter | 92,496 | Moderate-to-severe |
| Sundh 2015 [82] | Prospective obs. | Sweden | Multicenter | 373 | Moderate-to-severe |
| Tsiligianni 2016 (UNLOCK study) [83] | Cross-sectional | Netherlands | Multicenter | 2269 | Moderate-to-severe |
| Urwyler 2019 [84] | Retrospective obs. | Switzerland | Multicenter | 1247 | Moderate-to-severe |
| Vedel-Krogh 2016 (Copenhagen General Population Study) [85] | Prospective obs. | Denmark | Multicenter | 7225 | Moderate-to-severe |
| Vestbo 2017 (TRINITY trial) [89] | RCT | Global | Multicenter international | 2691 | Moderate-to-severe |
| Vestbo 2019 [90] | Cross-sectional | Global | Multicenter international | 1528 | Moderate-to-severe |
| Vogelmeier 2019 [91] | Retrospective obs. | US and UK | Multicenter | 15,364 (CPRD); 139,465 (Optum) | Moderate-to-severe |
| Wallace 2019 [92] | Retrospective obs. | US | Multicenter | 1505 | Moderate-to-severe |
| Watz 2016 (WISDOM trial) [93] | RCT | US | Multicenter | 2296 | Moderate-to-severe |
| Wei 2017 (TOLD study) [100] | Retrospective obs. | Taiwan | Multicenter | 1096 | Moderate-to-severe |
| Wei 2018 [101] | Retrospective obs. | China | Single center | 243 | Severe |
| Westerik 2017 [102] | Retrospective obs. | Netherlands | Single center | 14,603 | Moderate |
| Whalley 2019 (The Salford Lung study) [103] | RCT | UK | Single center | 400 | Moderate-to-severe |
| Wu 2018 [104] | Retrospective obs. | China | Multicenter | 744 | Moderate-to-severe |
| Yii 2019 [105] | Retrospective obs. | Singapore | Single center | 237 | Severe |
| Yohannes 2017 (ECLIPSE study) [106] | Prospective obs. | Global | Multicenter international | 2059 | Moderate-to-severe |
| Yun 2018 (ECLIPSE + COPDGene studies) [111] | Cross-sectional and longitudinal | Global | Multicenter international | 3448 | Moderate-to-severe |
| Zeiger 2018 [112] | Retrospective obs. | US | Multicenter | 7245 | Moderate-to-severe |

Study characteristics were captured from the primary publication

*obs* observational, *RCT* randomized clinical trial

Supplementary Table 4 Clinical characteristics of the patients assessed across the included studies

| Study | Mean age (years) | Male, % | Mean BMI (kg/m^2^) | Prior exacerbations, % | | | | GOLD severity, % | | | | | | | |
| --- | --- | --- | --- | --- | --- | --- | --- | --- | --- | --- | --- | --- | --- | --- | --- |
|  |  |  |  | 0 | ≥1 | ≥2 | 0–1 | Mild | Moderate | Severe | Very severe | A | B | C | D |
| Adir 2018 [1] | 70.8 | 63.8 | 28.4 | - | - | - | - | - | - | - | - | - | - | - | - |
| Alexopoulos 2015 (GOLDEN study) [2] | 68^a^ | 71.3 | 27.5^a^ | - | - | - | - | 19.3 | 35.4 | 25.6 | 19.6 | - | - | - | - |
| Annavarapu 2018 [3] | 71.4 | 39.7 | - | - | - | - | - | - | - | - | - | - | - | - | - |
| Bade 2019 [4] | 65.5 | 96.3 | - | - | - | - | - | - | - | - | - | - | - | - | - |
| Bafadhel 2018 [5] | 63 | 65 | 26.1 | - | - | - | - | - | 18 | 58 | 23 | - | - | - | - |
| Bartels 2018 [6]^b^ | 66.2 | 64.8 | 26.7 | - | - | - | - | - | - | - | - | - | - | - | - |
| Baumeler 2016 (PROMISE-COPD study) [8] | 67^a^ | 70.2 | 26.6 | - | - | - | - | - | 47.5 | 34 | 15.8 | - | - | - | - |
| Bodduluri 2018 (COPDGene study) [45] | 59.9 | 54.1 | 28.3 | - | - | - | - | 8.8 | 21.5 | 12.9 | 1.2 | - | - | - | - |
| Calverley 2018 (TIOSPIR study) [14] | 65.2 | 67.8 | 26.1 | 44.2 | 55.7 | 25.5 | 74.4 | 42.3 | | 43.1 | 13.3 | - | - | - | - |
| Chapman 2018 (SUNSET trial) [17] | 65.3 | 70.6 | 28 | 65.9 | - | - | 100 | - | 69.8 | 29.9 | - | - | - | - | - |
| Couillard 2017 [18] | 71.4 | 51.5 | - | - | - | - | - | 7.8 | 47.9 | 35.3 | 9 | - | - | - | - |
| de Miguel-Díez 2019 [20] | 75.2 | 83.5 | - | - | - | - | - | - | - | - | - | - | - | - | - |
| Eklöf 2020 (e-publication, 2019) [21] | 69^a^ | 44.8 | 25^a^ | - | - | - | - | - | - | - | - | - | - | - | - |
| Emura 2019 [22] | 71.9 | 79.6 | - | - | - | - | - | - | 33.5 | 44 | 18.3 | - | - | - | - |
| Engel 2017 (REDUCE study) [23] | 69.8 | 60.45 | - | - | - | - | - | 0.3 | 13.3 | 32.6 | 53.8 | - | - | - | - |
| Erol 2018 [24] | 65 | 88.9 | - | - | 61.3 | - | - | - | - | - | - | 24.9 | 20.9 | 12.9 | 41.3 |
| Estirado 2018 [25] | 71.7 | 83.6 | 27.3 | - | - | - | - | - | - | - | - | - | - | - | - |
| Ferguson 2018 (KRONOS study) [26] | 65.1 | 70 | 26.2 | 74.4 | 25.6 | 6.6 | 93.4 | 0.15 | 49.22 | 42.88 | 7.75 | - | 87.82 | - | 10.95 |
| Fuhrman 2017 [27] | 72.6 | 61.4 | - | - | - | - | - | - | - | - | - | - | - | - | - |
| Han 2017 (SPIROMICS study) [28] | 66 | 57.1 | 27.7 | - | 24.1 | 9.6 | - | 24.8 | 44.7 | 22.6 | 7.9 | - | - | - | - |
| Han 2018 [33] | 70.4 | 93.7 | 23.3 | - | - | - | - | - | - | - | - | - | - | - | - |
| Huang 2018 [34] | 71 | 94 | 23.1 | - | - | - | - | - | - | - | - | - | - | - | - |
| Jo 2018 [35] | 71.2 | 97.6 | 23.1 | - | - | - | - | 16.9 | | 59.4 | 20.3 | 6.7 | - | - | - |
| Jo 2018b [36]^b^ | 65.7 | 95.6 | 22.4 | - | - | - | - | 100 | | - | - | - | - | - | - |
| Jung 2015 [38] | 69.2 | 93.2 | 22.1 | - | - | - | - | - | - | - | - | 22.9 | 38.1 | 11 | 28 |
| Kim 2017 [40] | 68.3 | 90.9 | 23 | - | - | - | - | - | - | - | - | - | - | - | - |
| Ko 2019 [47] | 74.9 | 96.2 | 21.1 | - | - | - | - | - | - | - | - | - | - | - | - |
| Kobayashi 2018 [48] | 75.1 | 90.3 | 23 | - | - | - | - | 21.2 | 51.4 | 19.7 | 7.7 | 59.9 | 30.4 | 4 | 5.7 |
| Krachunov 2017 [49] | 68.5 | 71.1 | - | - | - | - | - | - | - | - | - | 9.2 | 15.7 | 24.5 | 50.6 |
| Krachunov 2018 [50] | 67 | 72.7 | 28 | - | - | - | - | 9.9 | 48.9 | 29.9 | 13.3 | 27.7 | 18.1 | 16.1 | 38.1 |
| Lau 2017 [51] | - | 44.5 | - | - | - | - | - | - | - | - | - | - | - | - | - |
| Lee 2019 [52] | 68.1 | 92.4 | 23.4 | 79.5 | 18.4 | 8.4 | 89.5 | - | - | - | - | 25.6 | 59.4 | 2.3 | 10.1 |
| Liu 2015 [54] | 71.5^a^ | 81.8 | 22.4 | - | - | - | - | 12 | 16.5 | 40.3 | 31.3 | - | - | - | - |
| MacDonald 2019 [55] | 72.6 | 59.6 | 26.2 | - | - | - | - | - | - | - | - | - | - | - | - |
| Make 2015 [56] | 63.1 | 64.21 | 26.7 | - | 100 | 39.9 | 60 | - | 20.7 | 56.4 | 22.2 | - | - | - | - |
| Marçôa 2018 [57] | 69 | 87.5 | 25.3 | - | - | - | - | - | - | - | - | - | - | - | - |
| Margüello 2016 [58] | 71.2 | 78.4 | 28.9 | 36.1 | - | 37.4 | 62.6 | 8.33 | 48.11 | 21.11 | 2.77 | - | - | - | - |
| McGarvey 2015 [59] | 69.5 | 50.9 | 27.2 | - | - | - | - | 17.5 | 52 | 25.3 | 5.2 | - | - | - | - |
| Montserrat-Capdevila 2015 [60] | 68.4 | 75 | - | - | - | - | - | 50.8 | 35.3 | 9.4 | 4.4 | - | - | - | - |
| Montserrat-Capdevila 2016 [61] | 69.5 | 73.2 | 29.5 | - | - | - | - | - | - | - | - | - | 27.1 | 50.8 | 22.1 |
| Müllerová 2019 (HO-17-18395 study) [62] | 71 | 42.5 | 29.3 | - | - | - | - | - | - | - | - | - | - | - | - |
| Orea-Tejeda 2018 [63] | 75 | 43.6 | 29 | - | - | - | - | - | - | - | - | - | - | - | - |
| Papi 2018 (TRIBUTE trial) [64] | 64.4 | 72 | 26.1 | - | 100 | 19 | 80.8 | - | - | 79.5 | 20.5 | - | - | - | 100 |
| Pascoe 2019 (IMPACT trial) [65] | 65.3 | 66 | - | <1 | 99.9 | 55 | 46 | <1 | 36 | 48 | 16 | - | - | - | - |
| Pasquale 2016 [67] | 72.2 | 45.75 | - | - | - | - | - | - | - | - | - | - | - | - | - |
| Pavlovic 2017 [68] | 66.9 | 67.8 | 25.5 | - | - | - | - | - | - | - | - | - | - | - | - |
| Pavord 2016 (INSPIRE, TRISTAN, SCO30002 trials) [69] | 68.1 | 76.7 | - | - | - | - | - | - | - | - | - | - | - | - | - |
| Pikoula 2019 [70] | - | 54.5 | - | - | - | - | - | 26.1 | 50.2 | 20.4 | 3.3 | - | - | - | - |
| Roche 2017 (FLAME trial) [71] | 64.5 | 75.5 | - | 0.059 | - | 19.37 | 80.59 | 0 | 33.4 | 58.1 | 7.6 | <1 | 24.5 | <1 | 74.8 |
| Rothnie 2018 [72] | 67 | 53.9 | 27 | - | - | - | - | - | - | - | - | - | - | - | - |
| Santibáñez 2016 [73] | 71.2 | 78.4 | - | 36.1 | - | 37.4 | 62.5 | 10.4 | 59.9 | 26.3 | 3.5 | - | - | - | - |
| Schuler 2018 (RIMTCORE study) [75] | 58 | 65.3 | 27 | - | - | - | - | - | 51.7 | 39.2 | 9.1 | - | - | - | - |
| Singh 2016 (TRILOGY trial) [76] | 63.6 | 75 | 26.3 | - | 100 | 19.8 | 80.2 | - | - | 77 | 23 | - | - | - | 100 |
| Søgaard 2016 [77] | - | 47 | - | - | - | - | - | - | - | - | - | - | - | - | - |
| Song 2018 [78] | 69.2 | 93.9 | 22.8 | - | - | - | - | - | - | - | - | - | - | - | - |
| Stanford 2018 (HO-11-732 study) [79] | 68.1 | 46 | - | - | - | - | - | - | - | - | - | - | - | - | - |
| Stanford 2019 [81] | 69 | 47.3 | - | - | - | - | - | - | - | - | - | - | - | - | - |
| Sundh 2015 [82] | 71.2 | 55.8 | - | - | - | - | - | - | - | 69.4 | 30.6 | - | - | - | - |
| Tsiligianni 2016 (UNLOCK study) [83] | 65.3 | 57.8 | - | - | - | - | - | 29.5 | 55.4 | 14 | 1.1 | 28.5 | 40.4 | 7.8 | 23.2 |
| Urwyler 2019 [84] | 66.4 | 60.4 | 26.5 | - | - | - | - | 3.63 | 31.9 | 29.3 | 10.8 | - | - | - | - |
| Vedel-Krogh 2016 (Copenhagen General Population Study) [85] | 64^a^ | 50.0 | 25.0 | - | - | - | - | 46.0 | 45.0 | 9.0 | - | - | - | - | - |
| Vestbo 2017 (TRINITY trial) [89] | 63.2 | 76 | 26.3 | - | 100 | 20.6 | 79.4 | - | - | 79 | 21 | - | - | - | 100 |
| Vestbo 2019 [90] | 65.7 | 68.2 | 26.7 | - | - | - | - | - | - | - | - | 12 | 61.2 | 0.9 | 26 |
| Vogelmeier 2019 [91] | 71.8 | 51.5 | - | - | - | - | - | - | - | - | - | - | - | - | - |
| Wallace 2019 [92] | 69 | 50 | - | - | - | - | - | 22 | 54.7 | 21.2 | 2.1 | - | - | - | - |
| Watz 2016 (WISDOM trial) [93] | 63.8 | 82.8 | 25.1 | - | - | - | - | <1 | <1 | 61 | 38 | - | - | - | - |
| Wei 2017 (TOLD study) [100] | - | 93.62 | - | 67.2 | - | 13.17 | 82.04 | - | - | - | - | 14.85 | 35.82 | 9.07 | 40.22 |
| Wei 2018 [101] | 67.9 | 92 | 23.2 | - | - | - | - | 7.4 | 33.7 | 41.6 | 17.3 | - | - | - | - |
| Westerik 2017 [102] | 66.5 | 53.1 | - | - | - | 6.12 | - | - | - | - | - | - | - | - | - |
| Whalley 2019 (The Salford Lung study) [103] | 66.16 | 53.25 | - | 31.25 | 23.25 | 45.5 | - | - | - | - | - | - | - | - | - |
| Wu 2018 [104] | 67.4^a^ | 77 | 24.5^a^ | - | - | - | - | - | - | - | - | - | - | - | - |
| Yii 2019 [105] | 75 | 93.7 | 20.9 | - | - | - | - | - | - | - | - | - | - | - | - |
| Yohannes 2017 (ECLIPSE study) [106] | 63.4 | 65.3 | 26.6 | - | - | - | - | - | 100 | | - | - | - | - | - |
| Yun 2018 (ECLIPSE + COPDGene studies) [111] | 65.81 | 63.18 | 27.41 | - | - | - | - | - | 50.17 | 37.22 | 12.57 | - | - | - | - |
| Zeiger 2018 [112] | 71.5 | 57.1 | - | - | - | - | - | - | - | - | - | - | - | - | - |

^a^Median value; ^b^Weighted mean value. *BMI*, body mass index, *GOLD*, Global Initiative for Obstructive Lung Disease

**References**

1. Adir Y, Hakrush O, Shteinberg M, Schneer S, Agusti A. Circulating eosinophil levels do not predict severe exacerbations in COPD: a retrospective study. ERJ Open Research. 2018;4:00022–02018.

2. Alexopoulos EC, Malli F, Mitsiki E, Bania EG, Varounis C, Gourgoulianis KI. Frequency and risk factors of COPD exacerbations and hospitalizations: a nationwide study in Greece (Greek Obstructive Lung Disease Epidemiology and health ecoNomics: GOLDEN study). Int J Chron Obstruct Pulmon Dis. 2015;10:2665–2674.

3. Annavarapu S, Goldfarb S, Gelb M, Moretz C, Renda A, Kaila S. Development and validation of a predictive model to identify patients at risk of severe COPD exacerbations using administrative claims data. Int J Chron Obstruct Pulmon Dis. 2018;13:2121–2130.

4. Bade BC, DeRycke EC, Ramsey C, Skanderson M, Crothers K, Haskell S, Bean-Mayberry B, Brandt C, Bastian LA, Akgün KM. Sex differences in veterans admitted to the hospital for chronic obstructive pulmonary disease exacerbation. Ann Am Thorac Soc. 2019;16:707–714.

5. Bafadhel M, Peterson S, De Blas MA, Calverley PM, Rennard SI, Richter K, Fagerås M. Predictors of exacerbation risk and response to budesonide in patients with chronic obstructive pulmonary disease: a post-hoc analysis of three randomised trials. Lancet Respir Med. 2018;6:117–126.

6. Bartels W, Adamson S, Leung L, Sin DD, van Eeden SF. Emergency department management of acute exacerbations of chronic obstructive pulmonary disease: factors predicting readmission. Int J Chron Obstruct Pulmon Dis. 2018;13:1647–1654.

7. Van Eeden SF, Bartels W, Adamson SL, Leung L, Sin DD. Emergency department management of acute exacerbations of chronic obstructive pulmonary disease (AECOPD): factors predicting readmission [abstract]. Am J Respir Crit Care Med. 2018;197.

8. Baumeler L, Papakonstantinou E, Milenkovic B, Lacoma A, Louis R, Aerts JG, Welte T, Kostikas K, Blasi F, Boersma W, et al. Therapy with proton-pump inhibitors for gastroesophageal reflux disease does not reduce the risk for severe exacerbations in COPD. Respirology. 2016;21:883–890.

9. Egli A, Mandal J, Schumann DM, Roth M, Thomas B, Lorne Tyrrell D, Blasi F, Kostikas K, Boersma W, Milenkovic B, et al. IFNΛ3/4 locus polymorphisms and IFNΛ3 circulating levels are associated with COPD severity and outcomes. BMC Pulm Med. 2018;18:51.

10. Mandal J, Roth M, Papakonstantinou E, Tamm M, Boeck L, Scherr A, Rakic J, Louis R, Milencovic B, Boersma W, et al. Irisin is associated with severe exacerbations of COPD independently of lung function, comorbidities and exercise capacity [abstract]. Eur Respir J. 2015;46 (suppl 59).

11. Papakonstantinou E, Bonovolias I, Roth M, Tamm M, Schumann D, Baty F, Louis R, Milenkovic B, Boersma W, Stieltjes B, et al. Serum levels of hyaluronic acid are associated with COPD severity and predict survival. Eur Respir J. 2019;53:1801183.

12. Mandal J, Malla B, Steffensen R, Costa L, Egli A, Trendlenburg M, Blasi F, Kostikas K, Welte T, Torres A, et al. Mannose-binding lectin protein and its association to clinical outcomes in COPD: a longitudinal study [abstract]. Kardiovask Med. 2016;19(5):86 S.

13. Papakonstantinou E, Bonovolias I, Karakioulakis G, Tamm M, Louis R, Milenkovic B, Boersma W, Kostikas K, Blasi F, Aerts J, et al. Serum hyaluronidase-1 is increased in COPD exacerbations and is associated with COPD severity and outcomes [abstract]. Eur Respir J. 2018;52 (suppl 62).

14. Calverley PM, Anzueto AR, Dusser D, Mueller A, Metzdorf N, Wise RA. Treatment of exacerbations as a predictor of subsequent outcomes in patients with COPD. Int J Chron Obstruct Pulmon Dis. 2018;13:1297–1308.

15. Calverley PM, Tetzlaff K, Dusser D, Wise RA, Mueller A, Metzdorf N, Anzueto A. Determinants of exacerbation risk in patients with COPD in the TIOSPIR study. Int J Chron Obstruct Pulmon Dis. 2017;12:3391–3405.

16. McGarvey L, Calverley PM, Metzdorf N, Mueller A, Wise RA, Anzueto A, Dusser D. Productive cough as a predictor of mortality, exacerbations and cardiac events in the Tiospir® trial [abstract]. Respirology. 2016;21(S3).

17. Chapman KR, Hurst JR, Frent SM, Larbig M, Fogel R, Guerin T, Banerji D, Patalano F, Goyal P, Pfister P, et al. Long-term triple therapy de-escalation to indacaterol/glycopyrronium in patients with chronic obstructive pulmonary disease (SUNSET): a randomized, double-blind, triple-dummy clinical trial. Am J Respir Crit Care Med. 2018;198:329–339.

18. Couillard S, Larivée P, Courteau J, Vanasse A. Eosinophils in COPD exacerbations are associated with increased readmissions. Chest. 2017;151:366–373.

19. Crisafulli E, Torres A, Huerta A, Méndez R, Guerrero M, Martinez R, Liapikou A, Soler N, Sethi S, Menéndez R. C-reactive protein at discharge, diabetes mellitus and ≥1 hospitalization during previous year predict early readmission in patients with acute exacerbation of chronic obstructive pulmonary disease. COPD. 2015;12:311–320.

20. de Miguel-Díez J, Hernández-Vázquez J, López-de-Andrés A, Álvaro-Meca A, Hernández-Barrera V, Jiménez-García R. Analysis of environmental risk factors for chronic obstructive pulmonary disease exacerbation: A case-crossover study (2004-2013). PLoS One. 2019;14:e0217143.

21. Eklöf J, Sørensen R, Ingebrigtsen TS, Sivapalan P, Achir I, Boel JB, Bangsborg J, Ostergaard C, Dessau RB, Jensen US, et al. Pseudomonas aeruginosa and risk of death and exacerbations in patients with chronic obstructive pulmonary disease: an observational cohort study of 22 053 patients. Clin Microbiol Infect. 2020;26:227–234.

22. Emura I, Usuda H, Satou K. Appearance of large scavenger receptor A-positive cells in peripheral blood: A potential risk factor for severe exacerbation of chronic obstructive pulmonary disease. Pathol Int. 2019;69:187–192.

23. Engel B, Schindler C, Leuppi JD, Rutishauser J. Predictors of re-exacerbation after an index exacerbation of chronic obstructive pulmonary disease in the REDUCE randomised clinical trial. Swiss Med Wkly. 2017;147:w14439.

24. Erol S, Sen E, Gizem Kilic Y, Yousif A, Akkoca Yildiz O, Acican T, Saryal S. Does the 2017 revision improve the ability of GOLD to predict risk of future moderate and severe exacerbation? Clin Respir J. 2018;12:2354–2360.

25. Estirado C, Ceccato A, Guerrero M, Huerta A, Cilloniz C, Vilaró O, Gabarrús A, Gea J, Crisafulli E, Soler N, Torres A. Microorganisms resistant to conventional antimicrobials in acute exacerbations of chronic obstructive pulmonary disease. Respir Res. 2018;19:119.

26. Ferguson GT, Rabe KF, Martinez FJ, Fabbri LM, Wang C, Ichinose M, Bourne E, Ballal S, Darken P, DeAngelis K, et al. Triple therapy with budesonide/glycopyrrolate/formoterol fumarate with co-suspension delivery technology versus dual therapies in chronic obstructive pulmonary disease (KRONOS): a double-blind, parallel-group, multicentre, phase 3 randomised controlled trial. Lancet Respir Med. 2018;6:747–758.

27. Fuhrman C, Moutengou E, Roche N, Delmas MC. Prognostic factors after hospitalization for COPD exacerbation. Rev Mal Respir. 2017;34:1–18.

28. Han MK, Quibrera PM, Carretta EE, Barr RG, Bleecker ER, Bowler RP, Cooper CB, Comellas A, Couper DJ, Curtis JL, et al. Frequency of exacerbations in patients with chronic obstructive pulmonary disease: an analysis of the SPIROMICS cohort. Lancet Respir Med. 2017;5:619–626.

29. Diamond M, Zhao H, Armstrong HF, Morrison M, Bailey KL, Carretta EE, Criner GJ, Han MK, Bleeker E, Cooper CB, et al. Anxiety and depression, either alone or in combination, are associated with respiratory exacerbations in smokers with and without COPD [abstract]. Am J Respir Crit Care Med. 2017;195.

30. Putcha N, Barr RG, Han M, Woodruff PG, Bleecker ER, Kanner RE, Martinez FJ, Tashkin DP, Rennard SI, Breysse P, et al. Understanding the impact of passive smoke exposure on outcomes in COPD [abstract]. Am J Respir Crit Care Med. 2015;191.

31. Keene JD, Jacobson S, Kechris K, Kinney GL, Foreman MG, Doerschuk CM, Make BJ, Curtis JL, Rennard SI, Barr RG, et al. Biomarkers predictive of exacerbations in the SPIROMICS and COPDGene cohorts. Am J Respir Crit Care Med. 2017;195:473–481.

32. Paul GG, Putcha N, Wise RA, O'Neal W, Dransfield M, Woodruff PG, Comellas AP, Drummond MB, Lambert A, Paulin LM, et al. Lower serum IgA is associated with COPD exacerbation risk: Spiromics and COPD gene [abstract]. Am J Respir Crit Care Med. 2017;195.

33. Han MZ, Hsiue TR, Tsai SH, Huang TH, Liao XM, Chen CZ. Validation of the GOLD 2017 and new 16 subgroups (1A-4D) classifications in predicting exacerbation and mortality in COPD patients. Int J Chron Obstruct Pulmon Dis. 2018;13:3425–3433.

34. Huang TH, Hsiue TR, Lin SH, Liao XM, Su PL, Chen CZ. Comparison of different staging methods for COPD in predicting outcomes. Eur Resp J. 2018;51:1700577.

35. Jo YS, Yoon HI, Kim DK, Yoo CG, Lee CH. Comparison of COPD Assessment Test and Clinical COPD Questionnaire to predict the risk of exacerbation. Int J Chron Obstruct Pulmon Dis. 2018;13:101–107.

36. Jo YS, Kim YH, Lee JY, Kim K, Jung KS, Yoo KH, Rhee CK. Impact of BMI on exacerbation and medical care expenses in subjects with mild to moderate airflow obstruction. Int J Chron Obstruct Pulmon Dis. 2018;13:2261–2269.

37. Lee H, Rhee CK, Lee BJ, Choi DC, Kim JA, Kim SH, Jeong Y, Kim TH, Chon GR, Jung KS, et al. Impacts of coexisting bronchial asthma on severe exacerbations in mild-to-moderate COPD: results from a national database. Int J Chron Obstruct Pulmon Dis. 2016;11:775–783.

38. Jung YH, Lee DY, Kim DW, Park SS, Heo EY, Chung HS, Kim DK. Clinical significance of laryngopharyngeal reflux in patients with chronic obstructive pulmonary disease. Int J Chron Obstruct Pulmon Dis. 2015;10:1343–1351.

39. Lee DY, Jung YH, Park SS, Chung HS, Heo EY, Kim DK. The clinical significance of laryngopharyngeal reflux in patients with chronic obstructive pulmonary disease [abstract]. Eur Respir J. 2015;46 (suppl 59).

40. Kim J, Kim WJ, Lee CH, Lee SH, Lee MG, Shin KC, Yoo KH, Lee JH, Lim SY, Na JO, et al. Which bronchodilator reversibility criteria can predict severe acute exacerbation in chronic obstructive pulmonary disease patients? Respir Res. 2017;18:107.

41. Kim J, Kim W, Lee C-H, Lee S, Lee M-G, Shin K-C, Yoo K, Lee J-H, Lim S, Na J, et al. Severe acute exacerbation using various bronchodilator reversibility criteria in chronic obstructive pulmonary disease patients [abstract]. Am J Respir Crit Care Med. 2017;195.

42. Kim V, Zhao H, Regan E, Han MK, Make BJ, Crapo JD, Jones PW, Curtis JL, Silverman EK, Criner GJ, COPDGene Investigators. The St. George's Respiratory Questionnaire definition of chronic bronchitis may be a better predictor of COPD exacerbations compared with the classic definition. Chest. 2019;156:685–695.

43. Iyer AS, Bhatt SP, Dransfield M, Kinney G, Holm K, Wamboldt FS, Hanania N, Martinez C, Regan E, Foreman MG, et al. Psychological distress prospectively predicts severe exacerbations in smokers with and without airflow limitation - a longitudinal follow-up study of the COPDGene cohort [abstract]. Am J Respir Crit Care Med. 2017;195.

44. Abston E, Comellas A, Reed RM, Kim V, Wise RA, Brower R, Fortis S, Beichel R, Bhatt S, Zabner J, et al. Higher BMI is associated with higher expiratory airflow normalised for lung volume (FEF25-75/FVC) in COPD. BMJ Open Respir Res. 2017;4:e000231.

45. Bodduluri S, Puliyakote ASK, Gerard SE, Reinhardt JM, Hoffman EA, Newell JD, Jr., Nath HP, Han MK, Washko GR, San José Estepar R, et al. Airway fractal dimension predicts respiratory morbidity and mortality in COPD. J Clin Invest. 2018;128:5374-5382.

46. Kim V, Zhao H, Criner GJ, Jones PW, Regan E, Silverman EK, Make BJ, Curtis J, Crapo JD. The St. Georges Respiratory Questionnaire definition of chronic bronchitis is a better predictor of exacerbations than the classic definition in long-term follow-up in COPD patients [abstract]. Am J Respir Crit Care Med. 2017;195.

47. Ko FWS, Chan KP, Ngai J, Ng SS, Yip WH, Ip A, Chan TO, Hui DSC. Blood eosinophil count as a predictor of hospital length of stay in COPD exacerbations. Respirology. 2019;25:259–266.

48. Kobayashi S, Hanagama M, Ishida M, Sato H, Ono M, Yamanda S, Yamada M, Aizawa H, Yanai M. Clinical characteristics and outcomes in Japanese patients with COPD according to the 2017 GOLD classification: the Ishinomaki COPD Network Registry. Int J Chron Obstruct Pulmon Dis. 2018;13:3947–3955.

49. Krachunov II, Kyuchukov NH, Ivanova ZI, Yanev NA, Hristova PA, Borisova ED, Popova TP, Pavlov PS, Nikolova PT, Ivanov YY. Impact of air pollution and outdoor temperature on the rate of chronic obstructive pulmonary disease exacerbations. Folia Med (Plovdiv). 2017;59:423–429.

50. Krachunov I, Kyuchukov N, Ivanova Z, Yanev NA, Hristova PA, Pavlov P, Glogovska P, Popova T, Ivanov YY. Stability of frequent exacerbator phenotype in patients with chronic obstructive pulmonary disease. Folia Med (Plovdiv). 2018;60:536–545.

51. Lau CS, Siracuse BL, Chamberlain RS. Readmission After COPD Exacerbation Scale: determining 30-day readmission risk for COPD patients. Int J Chron Obstruct Pulmon Dis. 2017;12:1891–1902.

52. Lee SH, Lee JH, Yoon HI, Park HY, Kim TH, Yoo KH, Oh YM, Jung KS, Lee SD, Lee SW. Change in inhaled corticosteroid treatment and COPD exacerbations: an analysis of real-world data from the KOLD/KOCOSS cohorts. Respir Res. 2019;20:62.

53. Yoon HY, Park SY, Lee CH, Byun MK, Na JO, Lee JS, Lee WY, Yoo KH, Jung KS, Lee JH. Prediction of first acute exacerbation using COPD subtypes identified by cluster analysis. Int J Chron Obstruct Pulmon Dis. 2019;14:1389–1397.

54. Liu D, Peng SH, Zhang J, Bai SH, Liu HX, Qu JM. Prediction of short term re-exacerbation in patients with acute exacerbation of chronic obstructive pulmonary disease. Int J Chron Obstruct Pulmon Dis. 2015;10:1265–1273.

55. MacDonald MI, Osadnik CR, Bulfin L, Hamza K, Leong P, Wong A, King PT, Bardin PG. Low and high blood eosinophil counts as biomarkers in hospitalized acute exacerbations of COPD. Chest. 2019;156:92–100.

56. Make BJ, Eriksson G, Calverley PM, Jenkins CR, Postma DS, Peterson S, Östlund O, Anzueto A. A score to predict short-term risk of COPD exacerbations (SCOPEX). Int J Chron Obstruct Pulmon Dis. 2015;10:201–209.

57. Marçôa R, Rodrigues DM, Dias M, Ladeira I, Vaz AP, Lima R, Guimarães M. Classification of Chronic Obstructive Pulmonary Disease (COPD) according to the new Global Initiative for Chronic Obstructive Lung Disease (GOLD) 2017: Comparison with GOLD 2011. COPD. 2018;15:21–26.

58. Margüello MS, Garrastazu R, Ruiz-Nuñez M, Helguera JM, Arenal S, Bonnardeux C, León C, Miravitlles M, García-Rivero JL. Independent effect of prior exacerbation frequency and disease severity on the risk of future exacerbations of COPD: a retrospective cohort study. NPJ Prim Care Respir Med. 2016;26:16046.

59. McGarvey L, Lee AJ, Roberts J, Gruffydd-Jones K, McKnight E, Haughney J. Characterisation of the frequent exacerbator phenotype in COPD patients in a large UK primary care population. Respir Med. 2015;109:228–237.

60. Montserrat-Capdevila J, Godoy P, Marsal JR, Barbé F. Predictive model of hospital admission for COPD exacerbation. Respir Care. 2015;60:1288–1294.

61. Montserrat-Capdevila J, Godoy P, Marsal JR, Barbé F, Galván L. Risk factors for exacerbation in chronic obstructive pulmonary disease: a prospective study. Int J Tuberc Lung Dis. 2016;20:389–395.

62. Müllerová H, Hahn B, Simard EP, Mu G, Hatipoğlu U. Exacerbations and health care resource use among patients with COPD in relation to blood eosinophil counts. Int J Chron Obstruct Pulmon Dis. 2019;14:683–692.

63. Orea-Tejeda A, Navarrete-Peñaloza AG, Verdeja-Vendrell L, Jiménez-Cepeda A, González-Islas DG, Hernández-Zenteno R, Keirns-Davis C, Sánchez-Santillán R, Velazquez-Montero A, Puentes Rodríguez G. Right heart failure as a risk factor for severe exacerbation in patients with chronic obstructive pulmonary disease: Prospective cohort study. Clin Respir J. 2018;12:2635–2641.

64. Papi A, Vestbo J, Fabbri L, Corradi M, Prunier H, Cohuet G, Guasconi A, Montagna I, Vezzoli S, Petruzzelli S, et al. Extrafine inhaled triple therapy versus dual bronchodilator therapy in chronic obstructive pulmonary disease (TRIBUTE): a double-blind, parallel group, randomised controlled trial. Lancet. 2018;391:1076–1084.

65. Pascoe S, Barnes N, Brusselle G, Compton C, Criner GJ, Dransfield MT, Halpin DMG, Han MK, Hartley B, Lange P, et al. Blood eosinophils and treatment response with triple and dual combination therapy in chronic obstructive pulmonary disease: analysis of the IMPACT trial. Lancet Respir Med. 2019;7:745–756.

66. Lipson DA, Barnhart F, Brealey N, Brooks J, Criner GJ, Day NC, Dransfield MT, Halpin DMG, Han MK, Jones CE, et al. Once-daily single-inhaler triple versus dual therapy in patients with COPD. N Engl J Med. 2018;378:1671–1680.

67. Pasquale MK, Xu Y, Baker CL, Zou KH, Teeter JG, Renda AM, Davis CC, Lee TC, Bobula J. COPD exacerbations associated with the modified Medical Research Council scale and COPD assessment test among Humana Medicare members. Int J Chron Obstruct Pulmon Dis. 2016;11:111–121.

68. Pavlovic R, Stefanovic S, Lazic Z, Jankovic S. Factors associated with the rate of COPD exacerbations that require hospitalization. Turk J Med Sci. 2017;47:134–141.

69. Pavord ID, Lettis S, Locantore N, Pascoe S, Jones PW, Wedzicha JA, Barnes NC. Blood eosinophils and inhaled corticosteroid/long-acting beta-2 agonist efficacy in COPD. Thorax. 2016;71:118–125.

70. Pikoula M, Quint JK, Nissen F, Hemingway H, Smeeth L, Denaxas S. Identifying clinically important COPD sub-types using data-driven approaches in primary care population based electronic health records. BMC Med Inform Decis Mak. 2019;19:86.

71. Roche N, Chapman KR, Vogelmeier CF, Herth FJF, Thach C, Fogel R, Olsson P, Patalano F, Banerji D, Wedzicha JA. Blood eosinophils and response to maintenance chronic obstructive pulmonary disease treatment. Data from the FLAME trial. Am J Respir Crit Care Med. 2017;195:1189–1197.

72. Rothnie KJ, Müllerová H, Smeeth L, Quint JK. Natural history of chronic obstructive pulmonary disease exacerbations in a general practice-based population with chronic obstructive pulmonary disease. Am J Respir Crit Care Med. 2018;198:464–471.

73. Santibáñez M, Garrastazu R, Ruiz-Nuñez M, Helguera JM, Arenal S, Bonnardeux C, León C, García-Rivero JL. Predictors of hospitalized exacerbations and mortality in chronic obstructive pulmonary disease. PLoS One. 2016;11:e0158727.

74. Santibáñez M, Garrastazu R, Garcia-Rivero JL, Ruiz M, Arenal S, Helguera J, Bonnardeaux C, Leon C, Llorca J. Predictors of exacerbation frequency in chronic obstructive pulmonary disease [abstract]. Eur Resp J. 2015;46 (suppl 59).

75. Schuler M, Wittmann M, Faller H, Schultz K. Including changes in dyspnea after inpatient rehabilitation improves prediction models of exacerbations in COPD. Respir Med. 2018;141:87–93.

76. Singh D, Papi A, Corradi M, Pavlišová I, Montagna I, Francisco C, Cohuet G, Vezzoli S, Scuri M, Vestbo J. Single inhaler triple therapy versus inhaled corticosteroid plus long-acting β_2_-agonist therapy for chronic obstructive pulmonary disease (TRILOGY): a double-blind, parallel group, randomised controlled trial. Lancet. 2016;388:963–973.

77. Søgaard M, Madsen M, Løkke A, Hilberg O, Sørensen HT, Thomsen RW. Incidence and outcomes of patients hospitalized with COPD exacerbation with and without pneumonia. Int J Chron Obstruct Pulmon Dis. 2016;11:455–465.

78. Song JH, Lee CH, Um SJ, Park YB, Yoo KH, Jung KS, Lee SD, Oh YM, Lee JH, Kim EK, Kim DK. Clinical impacts of the classification by 2017 GOLD guideline comparing previous ones on outcomes of COPD in real-world cohorts. Int J Chron Obstruct Pulmon Dis. 2018;13:3473–3484.

79. Stanford RH, Nag A, Mapel DW, Lee TA, Rosiello R, Schatz M, Vekeman F, Gauthier-Loiselle M, Merrigan JFP, Duh MS. Claims-based risk model for first severe COPD exacerbation. Am J Manag Care. 2018;24:e45–e53.

80. Stanford RH, Nag A, Mapel DW, Lee TA, Rosiello R, Vekeman F, Gauthier-Loiselle M, Duh MS, Merrigan JF, Schatz M. Validation of a new risk measure for chronic obstructive pulmonary disease exacerbation using health insurance claims data. Ann Am Thorac Soc. 2016;13:1067–1075.

81. Stanford RH, Lau MS, Li Y, Stemkowski S. External validation of a COPD risk measure in a commercial and medicare population: the COPD treatment ratio. J Manag Care Spec Pharm. 2019;25:58–69.

82. Sundh J, Johansson G, Larsson K, Lindén A, Löfdahl CG, Sandström T, Janson C. The phenotype of concurrent chronic bronchitis and frequent exacerbations in patients with severe COPD attending Swedish secondary care units. Int J Chron Obstruct Pulmon Dis. 2015;10:2327–2334.

83. Tsiligianni I, Metting E, van der Molen T, Chavannes N, Kocks J. Morning and night symptoms in primary care COPD patients: a cross-sectional and longitudinal study. An UNLOCK study from the IPCRG. NPJ Prim Care Respir Med. 2016;26:16040.

84. Urwyler P, Hussein NA, Bridevaux PO, Chhajed PN, Geiser T, Grendelmeier P, Zellweger LJ, Kohler M, Maier S, Miedinger D, et al. Predictive factors for exacerbation and reexacerbation in chronic obstructive pulmonary disease: an extension of the Cox model to analyze data from the Swiss COPD cohort. Multidiscip Respir Med. 2019;14:7.

85. Vedel-Krogh S, Nielsen SF, Lange P, Vestbo J, Nordestgaard BG. Blood eosinophils and exacerbations in chronic obstructive pulmonary disease. The Copenhagen General Population Study. Am J Respir Crit Care Med. 2016;193:965–974.

86. Çolak Y, Afzal S, Marott JL, Nordestgaard BG, Vestbo J, Ingebrigtsen TS, Lange P. Prognosis of COPD depends on severity of exacerbation history: a population-based analysis. Respir Med. 2019;155:141–147.

87. Vedel-Krogh S, Nielsen SF, Lange P, Vestbo J, Nordestgard BG. Blood eosinophils and exacerbations in chronic obstructive pulmonary disease: the Copenhagen general population study [abstract]. Eur Respir J. 2015;46 (suppl 59).

88. Ingebrigtsen TS, Marott JL, Lange P, Hallas J, Nordestgaard BG, Vestbo J. Medically treated exacerbations in COPD by GOLD 1-4: A valid, robust, and seemingly low-biased definition. Respir Med. 2015;109:1562–1568.

89. Vestbo J, Papi A, Corradi M, Blazhko V, Montagna I, Francisco C, Cohuet G, Vezzoli S, Scuri M, Singh D. Single inhaler extrafine triple therapy versus long-acting muscarinic antagonist therapy for chronic obstructive pulmonary disease (TRINITY): a double-blind, parallel group, randomised controlled trial. Lancet. 2017;389:1919–1929.

90. Vestbo J, Vogelmeier CF, Small M, Siddall J, Fogel R, Kostikas K. Inhaled corticosteroid use by exacerbations and eosinophils: a real-world COPD population. Int J Chron Obstruct Pulmon Dis. 2019;14:853–861.

91. Vogelmeier CF, Kostikas K, Fang J, Tian H, Jones B, Morgan CL, Fogel R, Gutzwiller FS, Cao H. Evaluation of exacerbations and blood eosinophils in UK and US COPD populations. Respir Res. 2019;20:178.

92. Wallace AE, Kaila S, Bayer V, Shaikh A, Shinde MU, Willey VJ, Napier MB, Singer JR. Health care resource utilization and exacerbation rates in patients with COPD stratified by disease severity in a commercially insured population. J Manag Care Spec Pharm. 2019;25:205–217.

93. Watz H, Tetzlaff K, Wouters EFM, Kirsten A, Magnussen H, Rodriguez-Roisin R, Vogelmeier C, Fabbri LM, Chanez P, Dahl R, et al. Blood eosinophil count and exacerbations in severe chronic obstructive pulmonary disease after withdrawal of inhaled corticosteroids: a post-hoc analysis of the WISDOM trial. Lancet Respir Med. 2016;4:390–398.

94. Sajkov D, Calverley PMA, Tetzlaff K, Vogelmeier C, Fabbri LM, Magnussen H, Wouters EFM, Disse B, Finnigan H, Asijee GM, Watz H. Evaluating blood eosinophils and exacerbation history to predict inhaled corticosteroids response in COPD [abstract]. Respirology. 2017;22(S2).

95. Calverley PMA, Tetzlaff K, Vogelmeier C, Fabbri LM, Magnussen H, Wouters EFM, Disse B, Finnigan H, Asijee GM, Watz H, Laflamme K. Evaluating blood eosinophils and exacerbation history to predict ICS response in COPD [abstract]. Eur Respir J. 2016;48 (suppl 60).

96. Frith P, Watz H, Magnussen H, Rodriguez-Roisin R, Tetzlaff K, Haensel M, Mueller A, Vogelmeier C. ICS withdrawal and exacerbation risk by GOLD 2017 report: post hoc analysis of the WISDOM trial [abstract]. Respirology. 2019;24(S1):49.

97. Watz H, Magnussen H, Rodriguez-Roisin R, Tetzlaff K, Haensel M, Mueller A, Vogelmeier C. ICS withdrawal and exacerbation risk by GOLD 2017 report: post hoc analysis of the WISDOM trial [abstract]. Eur Respir J. 2018;52 (suppl 62).

98. Wouters EFM, Magnussen H, Rodriguez-Roisin R, Tetzlaff K, Bell S, Calverley PMA. Lung-function profile before and after the first moderate to severe exacerbation during the WISDOM study [abstract]. Pneumologie. 2017;71(S1).

99. Calverley PMA, Wouters EFM, Finngan H, Tetzlaff K, Magnussen H. Screennig eosinophil counts and risk of exacerbations after inhaled corticosteroid withdrawal in severe COPD [abstract]. Eur Respir J. 2016;48 (suppl 60).

100. Wei YF, Tsai YH, Wang CC, Kuo PH. Impact of overweight and obesity on acute exacerbations of COPD - subgroup analysis of the Taiwan Obstructive Lung Disease cohort. Int J Chron Obstruct Pulmon Dis. 2017;12:2723–2729.

101. Wei X, Ma Z, Yu N, Ren J, Jin C, Mi J, Shi M, Tian L, Gao Y, Guo Y. Risk factors predict frequent hospitalization in patients with acute exacerbation of COPD. Int J Chron Obstruct Pulmon Dis. 2018;13:121–129.

102. Westerik JAM, Metting EI, van Boven JFM, Tiersma W, Kocks JWH, Schermer TR. Associations between chronic comorbidity and exacerbation risk in primary care patients with COPD. Respir Res. 2017;18:31.

103. Whalley D, Svedsater H, Doward L, Crawford R, Leather D, Lay-Flurrie J, Bosanquet N. Follow-up interviews from The Salford Lung Study (COPD) and analyses per treatment and exacerbations. NPJ Prim Care Respir Med. 2019;29:20.

104. Wu Z, Yang D, Ge Z, Yan M, Wu N, Liu Y. Body mass index of patients with chronic obstructive pulmonary disease is associated with pulmonary function and exacerbations: a retrospective real world research. J Thorac Dis. 2018;10:5086–5099.

105. Yii ACA, Loh CH, Tiew PY, Xu H, Taha AAM, Koh J, Tan J, Lapperre TS, Anzueto A, Tee AKH. A clinical prediction model for hospitalized COPD exacerbations based on "treatable traits". Int J Chron Obstruct Pulmon Dis. 2019;14:719–728.

106. Yohannes AM, Mulerova H, Lavoie K, Vestbo J, Rennard SI, Wouters E, Hanania NA. The association of depressive symptoms with rates of acute exacerbations in patients with COPD: results from a 3-year longitudinal follow-up of the ECLIPSE cohort. J Am Med Dir Assoc. 2017;18:955–959.e6.

107. Wilke S, Jones PW, Mullerova H, Vestbo J, Tal-Singer R, Franssen FM, Agusti A, Bakke P, Calverley PM, Coxson HO, et al. One-year change in health status and subsequent outcomes in COPD. Thorax. 2015;70:420–425.

108. Müllerová H, Maselli DJ, Locantore N, Vestbo J, Hurst JR, Wedzicha JA, Bakke P, Agusti A, Anzueto A. Hospitalized exacerbations of COPD: risk factors and outcomes in the ECLIPSE cohort. Chest. 2015;147:999–1007.

109. Benson VS, Müllerová H, Vestbo J, Wedzicha JA, Patel A, Hurst JR, Evaluation of COPD Longitudinally to Identify Predictive Surrogate Endpoints (ECLIPSE) Investigators. Associations between gastro-oesophageal reflux, its management and exacerbations of chronic obstructive pulmonary disease. Respir Med. 2015;109:1147–1154.

110. Benson V, Müllerová H, Vestbo J, Wedzicha J, Patel A, Hurst J. Gastro-oesophageal reflux disease and exacerbations of COPD in a cohort of 2,135 COPD patients [abstract]. Eur Resp J. 2015;46 (suppl 59).

111. Yun JH, Lamb A, Chase R, Singh D, Parker MM, Saferali A, Vestbo J, Tal-Singer R, Castaldi PJ, Silverman EK, et al. Blood eosinophil count thresholds and exacerbations in patients with chronic obstructive pulmonary disease. J Allergy Clin Immunol. 2018;141:2037–2047.e10.

112. Zeiger RS, Tran TN, Butler RK, Schatz M, Li Q, Khatry DB, Martin U, Kawatkar AA, Chen W. Relationship of blood eosinophil count to exacerbations in chronic obstructive pulmonary disease. J Allergy Clin Immunol Pract. 2018;6:944–954.e945.
