# Supplementary material for: Prognostic risk factors for moderate-to-severe exacerbations in patients with chronic obstructive pulmonary disease: a systematic literature review
Source: Respir Res. 2022 Aug 23;23:213. doi: 10.1186/s12931-022-02123-5 (PMC9396841; doi:10.1186/s12931-022-02123-5)
Supplement: Supplementary file 2 — Additional file 2: Fig. S1. Sex (male vs female) as a risk factor for moderate-to-severe exacerbations. Fig. S2. Sex (male vs female) as a risk factor for severe exacerbations. [file 12931_2022_2123_MOESM2_ESM.docx]

Additional File 2

Supplementary Fig. 1 Sex (male vs female) as a risk factor for moderate-to-severe exacerbations


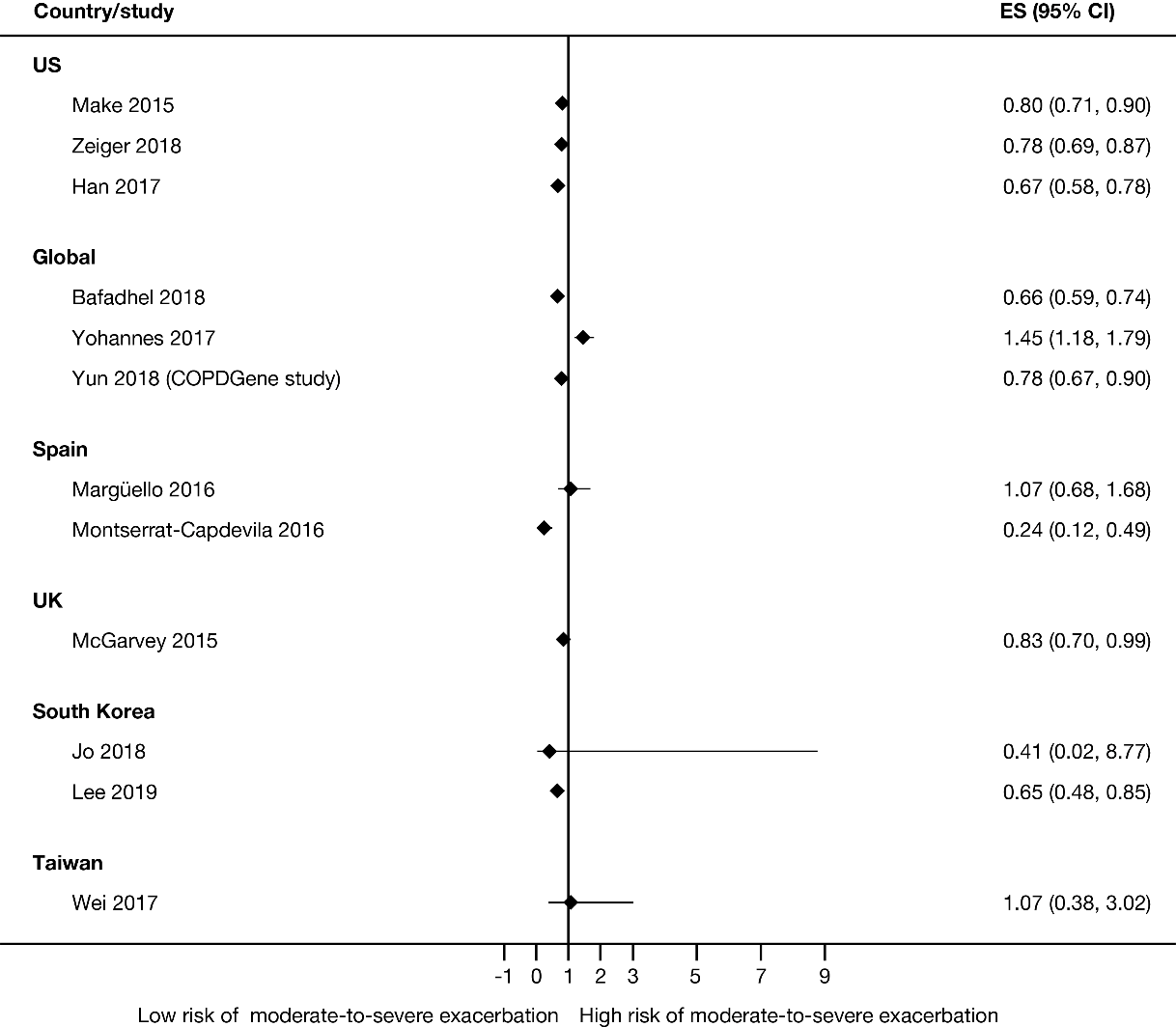


Yun 2018 included two studies; the study from which data were extracted (COPDGene or ECLIPSE) is listed in parentheses

*CI* confidence interval, *ES* effect size

Supplementary Fig. 2 Sex (male vs female) as a risk factor for severe exacerbations


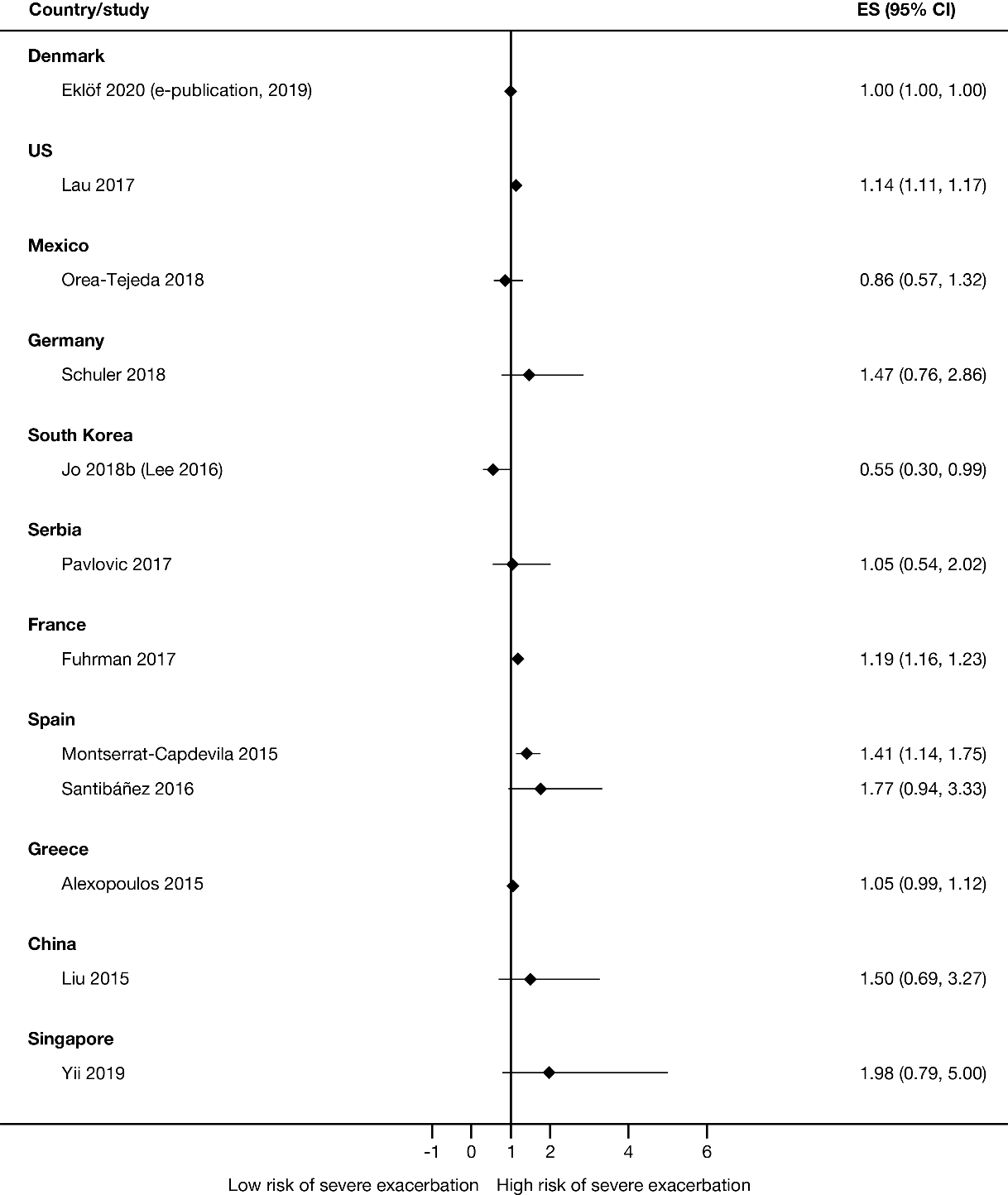


Where data have been extracted from a linked publication rather than the primary publication, the linked publication is listed in parentheses

*CI* confidence interval, *ES* effect size
